# Optical Fiber Sensor with Dynamically Responsive Cladding for Real-Time Breath Pattern Monitoring

**DOI:** 10.1021/acsomega.5c06598

**Published:** 2025-09-19

**Authors:** Pillalamarri Srikrishnarka, Jani Patrakka, Zhipei Sun, Nonappa

**Affiliations:** † Faculty of Engineering and Natural Sciences, 7840 Tampere University, Korkeakoulunkatu 6, FI-33720 Tampere, Finland; ‡ Department of Electronics and Nanoengineering, Aalto University, Maarintie 13, Espoo 02150, Finland

## Abstract

Rapid and real-time monitoring of humidity changes is critical, as they impact human health, material storage, and stability, industrial fabrication, microbial transmission, and agriculture. In recent years, optical fiber-based sensors have emerged as promising candidates for monitoring exhaled and inhaled breath humidity to assess respiratory rate, with the goal of supporting clinical diagnosis and patient care. Conventional multimode plastic optical fibers utilize a high refractive index core and lower refractive index cladding for effective total internal reflection (TIR) to achieve waveguiding. In contrast, here, we fabricated an optical fiber-based sensor with a higher refractive index (1.52) biopolymer-based cladding onto a lower refractive index (1.49) poly­(methyl methacrylate) (PMMA) core. We demonstrate that the cladding dynamically responds to a rapid humidity change by altering the refractive index and improving the TIR, allowing real-time humidity monitoring. Our results show that the sensitivity was characterized by a second-order polynomial fit, with a sensitivity of 0.18 dB/%RH (in the range of 40–70% RH) and an overall attenuation reduction of 9.4 dB at 70% RH. A proof-of-concept device, by ready integration of a fiber into a miniaturized platform, allowed for rapid and real-time exhaled breath humidity detection and differentiation of nasal and oral breathing patterns with a time resolution of 1.3 s. Notably, the device does not exhibit saturation under high humidity conditions, suggesting the robustness and enormous potential of optical fiber-based humidity sensors for long-term breath humidity monitoring, early detection of abnormal breath patterns, and deployment in next-generation healthcare monitoring.

## Graphical abstract

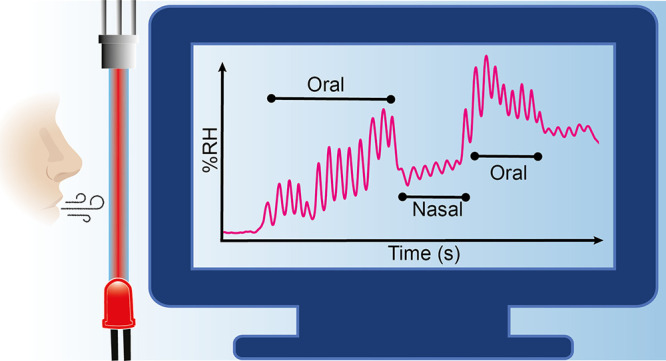

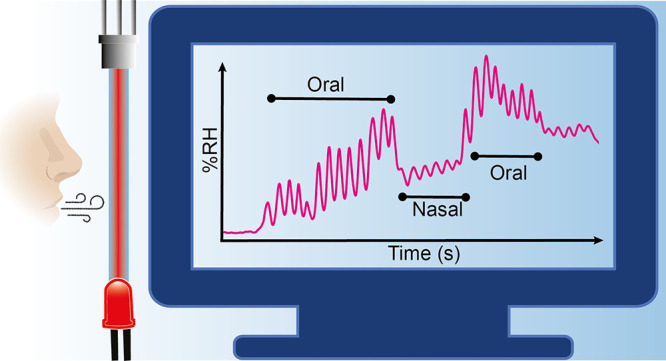

## 1 Introduction

Humidity is a vital environmental parameter warranting attention in numerous domains, including but not limited to agriculture,^1–3^ food storage,^4–6^ pharmaceuticals,^7–9^ climate,^10^ healthcare,^11–13^ automobiles,^14–16^ and semiconductor manufacturing.^17–19^ Exhaled breath analysis is a noninvasive measurement mode, with breath humidity being one of the parameters synchronous with breath, a key biomarker. It is usually influenced by the respiratory pattern, airway hydration, and metabolic activity of the epithelial cells.^20–22^ Noninvasive acquisition of these data enables the preemptive monitoring of hidden symptoms and pathological conditions. Therefore, affordable, rapid, specific, real-time, and sensitive sensors are needed. Conventional humidity sensors function based on capacitive,^23–26^ resistive,^27–31^ colorimetric,^29,32^ quartz crystal microbalance,^33–36^ surface acoustic waves,^37,38^ and field-effect transistors.^39,40^ These transduction techniques have been used to develop humidity sensors utilizing polymers,^41–43^ supramolecular systems,^44^ metal oxides,^45,46^ carbonaceous materials, and metal–organic frameworks. Resistive and capacitive sensors, although affordable and highly sensitive, depend on the operating temperature, display limited accuracy at saturating humidity, and suffer from material aging, which affects the sensor performance and power consumption. Furthermore, these sensors require sophisticated fabrication steps and are sensitive to electromagnetic interference. In recent years, optical fiber-based sensors have garnered considerable attention due to their immunity to electromagnetic interference. These sensors utilize Fabry-Pérot interferometry,^47–49^ fiber Bragg grating (FBG),^50,51^ Sagnac interference,^52–54^ and Mach–Zehnder interferometry.^55,56^

Conventional silica glass optical fibers (GOFs) show extremely low optical loss of ∼0.2 dB km^–1^ in the wavelength range 1500–1600 nm. Therefore, GOFs are extensively used for long-haul communication and high-speed data transmission.^57^ GOF-based sensors can sense strain, pressure, vibration, acceleration, temperature, and humidity in inextensible rigid structures such as roads, bridges, pipelines, and electrical grids.^58^ However, GOFs are brittle, inflexible, expensive, and difficult to handle. Therefore, incompatible for dense short-distance networks, soft robotics, consumer products, and emerging flexible devices involving large strains.^59^ Commercial plastic optical fibers (POFs) are lightweight and fatigue tolerant, making them attractive candidates for short-distance applications.^60^ However, GOFs and POFs lack intrinsic sensing abilities for environmental parameters such as humidity, and extensive postfabrication modifications are necessary to achieve sensing.^61^ Furthermore, fiber optic-based humidity sensors utilizing biopolymeric materials as a coating for commercial GOFs and POFs have been studied in the literature. Fu et al. have demonstrated that coating the tips of glass optical fibers with lignin allows humidity sensing with a high sensitivity of 4.7 dB.^62^ Yi et al. used a single-mode optical fiber coated with a gelatin film connected to a Mach–Zehnder interferometer, which showed rapid response and recovery times of 84 and 29 ms, respectively.^63^ The change in humidity altered the refractive index of the gelatin film, leading to a high sensitivity of 25 dB. Bao et al. inscribed Bragg’s grating on a single-mode glass optical fiber using a pulsed-mode femtosecond laser.^64^ The humidity-induced refractive index changes influenced the evanescent field of the guided cladding mode, which increased the light intensity, allowing rapid response and recovery time of 92 and 100 ms, respectively, for breath humidity measurements.^65^ Xia et al. prepared a no-core single-mode optical fiber incorporating polyvinylidene fluoride-hydroxyethyl cellulose hydrogel for relative humidity sensing (RH) with a sensitivity of 0.196 dB/%RH at a wavelength of 1310 nm.^66^ However, under extreme humidity conditions, hydrogels tend to undergo dehydration or swelling, limiting their application beyond a certain humidity range. Bariáin et al. studied an agarose hydrogel-coated tapered single-mode glass optical fiber and obtained a variation of 6.5 dB of the transmitted optical power with RH changes between 30% and 80% at 1310 nm.^67^ However, the response time was on the order of seconds to minutes. Arregui et al. investigated the humidity sensitivity of agarose, poly (2-hydroxyethyl methacrylate), poly-*N*-vinylpyrrolidone, and polyacrylamide with a response time of 90 s for the performing fibers.^68^ Gaston et al. used a side-polished single-mode optical fiber coated with a poly­(vinyl alcohol) with a power change of about 10 dB for RH between 70% and 90% at 1310 and 1550 nm.^69^ Xu et al. studied the silica optical fiber humidity sensor using evanescent-wave scattering caused by a porous sol–gel silica coating on the surface of a bent optical fiber core.^70^ Most of the studies utilized silica glass optical fibers with sophisticated fabrication and expensive light sources.

Recently, polysaccharides have been explored for optical fiber fabrication and short-distance applications due to their intrinsic sensing abilities resulting from their structural diversity, tunable refractive index, and surface chemistry.^71^ We have demonstrated that carboxymethylcellulose-based optical fibers enable humidity sensing and short-distance communication, albeit with their high optical loss and low mechanical performance.^72^ In another study, we have shown that methylcellulose-luminescent nanocluster composite optical fibers display a low attenuation coefficient of 1.49 dB cm^–1^.^73,74^ Furthermore, two- and three-component composite biopolymer optical fibers containing alginate and methylcellulose have been studied for rapid humidity sensing and enhanced performance in the near-infrared (NIR) region.^75^ More recently, we have demonstrated that fully biopolymer-based optical fibers allow quantitative humidity monitoring with high sensitivity of 0.30 dB/%RH.^76^ Despite their rapid humidity sensing properties, due to irregular morphology, integrating biopolymer-based optical fibers into a miniaturized device remains a challenge.

Alginates are linear polysaccharides obtained from the cell walls of brown algae, composed of β-(1–4)-D-mannuronic acid (M) and α-(1–4)-L-guluronic acid (G) units. Alginates are widely used in the food and biomedical industries, tissue engineering research, and for the removal of heavy metal ions.^77^ Alginates undergo cross-linking and gelation in the presence of divalent metal ions such as calcium (Ca^2+^), which is useful for preparing fibers.^75^ It has been demonstrated that metal ion-cross-linked alginate fibers exhibit humidity-dependent conductivity.^78^ The rapid absorption of water is attributed to the hydrophilic nature of alginate. More importantly, alginate in its solid state shows a high refractive index of 1.52.^79^ Traditionally, optical fibers contain a high refractive index core and a cladding with a constant refractive index lower than the refractive index of the core. The typical difference in refractive index between the core and cladding is very small, generally less than 1% in single-mode GOFs.^57–59^ In multimode POFs, the relative difference can range between 1 and 3%. The small difference is maintained to ensure total internal reflection for efficient light confinement and guiding, to reduce signal loss, to maintain modal dispersion control, and for numerical aperture tubing. Traditionally, POFs are composed of a poly­(methyl methacrylate) (PMMA, RI = 1.49) core and a fluorinated polymer cladding (RI = 1.37), allowing for total internal reflection. In contrast, we hypothesize that utilizing a biopolymer such as alginates with a higher refractive index as a cladding material in a PMMA fiber will enable rapid humidity sensing through dynamic modulation of the refractive index of the cladding. To test this hypothesis, we first used a COMSOL Multiphysics simulation of an optical fiber with a PMMA core (RI = 1.49) and hypothetical cladding with a refractive index of 1.52.

To experimentally validate our hypothesis, we coated the PMMA optical fiber (RI = 1.49) with alginate cladding (RI = 1.52) with varying thicknesses. Alginate is known for its rapid sorption and desorption of water molecules, which would dynamically modulate the refractive index of the cladding.^78^ Our results show that the fiber sensor exhibits a sensitivity of up to 0.18 dB/%RH in the 40–70% RH range, with a reduction in attenuation of ∼9.4 dB at 70% RH. The integration of the fiber into a miniaturized sensor enables rapid and real-time breath humidity detection, as well as the differentiation of nasal and oral breathing, with a time resolution of 1.3 s for noninvasive breath monitoring.

## 2 Experimental Section

### 2.1 Reagents and Materials

Sodium alginate (Alginic acid sodium salt) and calcium chloride (CaCl_2_, ≥ 96%) were purchased from Sigma-Aldrich. Sulfuric acid (H_2_SO_4_, 97%) was purchased from Merck, and ethyl acetate (99.9%) was procured from VWR Chemicals. All of the chemicals were used without any further purification. Ultrapure Milli-Q (18.2 MΩ·cm) water was used to prepare the alginate solution. Multimode poly­(methyl methacrylate) (PMMA, *d* ∼ 1 mm) having a core diameter of 980 μm and fluorinated polymer cladding of 10 μm thickness was procured from Farnell (OMPF1000).

### 2.2 Surface Treatment of the PMMA Optical Fiber

The commercial PMMA fibers were cut into 5 cm pieces by using a razor blade. The fiber tips were then polished using Thorlabs polishing sheets with grit sizes from 3 to 0.3 μm by moving the fiber tip in a figure-eight pattern on the sheet for 8 s. Fibers were then submerged in ethyl acetate for 45 s to remove the cladding. The treated fibers were wiped with lint-free tissue paper to remove residual cladding and then placed in a beaker containing a 3.0 M H_2_SO_4_ solution for 3 h at 60 °C.^65^ Subsequently, the fibers were immersed in Milli-Q ultrapure water (18.2 MΩ·cm) to remove any remaining acid moieties.

### 2.3 Preparation of Sodium Alginate Solution

An aqueous solution of sodium alginate (4 wt %) was prepared by adding 2.0 g of sodium alginate powder to a beaker containing 50 mL of ultrapure Milli-Q water (18.2 MΩ·cm). The mixture was stirred at room temperature (22–23 °C) for 4 h to ensure complete dissolution of the alginate. The solution was stored at +4 °C until further use.

### 2.4 Preparation of CaCl_2_ Solution

An aqueous solution of calcium chloride (5 wt %) was prepared by adding 10 g of CaCl_2_ to 200 mL of ultrapure Milli-Q water (18.2 MΩ·cm), followed by stirring until a clear solution was obtained. The solution was stored at +4 °C until further use.

### 2.5 Fabrication of the Alginate-Coated Optical Fiber (Alg@PMMA)

The alginate coating was applied to PMMA fibers utilizing the dip-coating method. The surface-treated fiber was immersed in alginate solution (4 wt %) for 10 s, followed by immersion in a CaCl_2_ solution for 2 min. Subsequently, the coated fiber was removed from the solution and allowed to dry overnight at room temperature (22 °C and 45% RH) to obtain PMMA fibers with alginate cladding, denoted as Alg@PMMA. The thickness of the cladding was tuned through repeated immersion of the coated fiber in an alginate solution, followed by subsequent immersion in a CaCl_2_ solution.

### 2.6 Humidity Sensing Measurement

To evaluate the sensing performance of the optical fibers, changes in transmission were assessed using an Avantes Starline (Avaspec-2048 L) spectrometer and an Ocean Optics (DH-2000-BAL) light source operating in the 300 to 1100 nm range with a 0.6 nm spectral resolution. The one end of the Alg@PMMA fiber was coupled with a 300 μm multimode optical fiber cable (Thorlabs, M12L01, Ø300 μm, 0.39 NA) with a Subminiature version A (SMA) connector, which was secured with a sleeve and a three-dimensional (3D)-printed ferrule, which was connected to the light source. The other end of the PMMA fiber was coupled to a 1000 μm multimode optical fiber and connected to the spectrometer. The Alg@PMMA optical fiber was placed inside a 3D-printed customized chamber with humidity control using a Linkam RH95 humidity controller with ± 0.5% stability and ± 1.5% sensor accuracy. The spectrometer integration time was adjusted to 4–8 ms to ensure a high photon count, and the resulting spectra were compared to the integration-time-adjusted baseline from the setup without the sample. Attenuation was calculated based on the equation below

1 $$a\mathrm{ttenuation}\;\left(\mathrm{dB}\right)=-10\log\left(\frac{I}{I_{o}}\right)$$

where *I* is the sample photon count after background correction and *I*_o_ is the (Background photon count multiplied by sample integration time) after background correction. Ambient environmental conditions were monitored by using a commercial RuuviTag humidity sensor.

### 2.7 COMSOL Multiphysics Simulation

To understand the effects of fiber coating on light transmission, a transparent light pipe model was used in the COMSOL Multiphysics simulation 6.1 software by modifying the hollow tube model. The simulation model comprises a concentric cylinder with an inner cylinder core diameter of 0.98 mm and a length of 5 cm. The dimensions of the outer cylinder were selected to align with the cladding thickness of Alg@PMMA fibers measured using scanning electron microscopy (SEM) imaging. The refractive index of the core was chosen as 1.49 based on the PMMA material provided in the COMSOL material database. The cladding’s refractive index was swept from 1.52 (the RI of dry alginate film) to 1.33 (the RI of pure water).^79^ Geometric ray optics with 1000 secondary waves and geometric normal-ray-boundary interactions were selected for simulation. The input light parameters were set at a 660 nm wavelength with an intensity of 1 mW. A refined finer mesh was implemented for the model evaluation. At the output of the fiber, the deposited ray power subnode was selected to measure the transmittance of the fiber. The transmittance was estimated by calculating the ratio of Deposited Ray Power to the Total Launch Power (1 mW).

### 2.8 Fabrication of the Miniaturized Sensor

The device comprises a 5 mm clear lens green LED operating at 520 nm, powered by an optical fiber laser emission module that can operate from 5 to 12 V and output a maximum current of 190 mA. The photodetector (PD) (BPX 65) was connected in reverse bias to the inverting output of the operational amplifier. When exposed to incident light from the optical fiber, the PD generates a photocurrent proportional to the light intensity. This current is further converted into voltage by the transimpedance amplifier circuit that includes a 1 MΩ feedback resistor in parallel to a 10 pF capacitor. This helps mitigate the high-frequency noise and prevents oscillations. The amplified signal is directly fed to the A0 analog pin of the Arduino Nano for data acquisition. The data were processed using a low-pass Butterworth filter to mitigate noise.

### 2.9 Characterization of Alg@PMMA Fibers

Fourier-transform infrared (FT-IR) spectra of the fibers were measured by using a PerkinElmer Spectrum Two IR spectrometer operated in the attenuated total reflection (ATR) mode. For scanning electron microscopy (SEM) imaging, the fibers were placed onto aluminum stubs by using carbon tape. The fibers were sputter-coated with Pt/Pd (thickness of ∼8 nm) using a Leica EM ACE600 high-vacuum sputter coater. The imaging was performed using a JEOL, JSM-IT500 FESEM operating at 5 kV. A Zeiss Axio Scope A1 microscope was used for polarizing optical microscopy (POM) imaging. Bright-field images were used for measuring fiber diameters with Zen 2 Blue Edition software. The cladding thickness was calculated using fiber samples in triplicate. The average thickness was 57 ± 13 μm, 180 ± 10 μm, and 330 ± 40 μm, respectively, for one-, two-, and three-cycle alginate coating and are denoted as Alg@PMMA-60, Alg@PMMA-170, and Alg@PMMA-370.

## 3 Results and Discussion

### 3.1 Fiber Morphology and Characterization

We have used a PMMA optical fiber (RI ∼ 1.49) with a core diameter of 980 and 10 μm fluorinated cladding (RI ∼ 1.37). The original fluorinated cladding of the PMMA optical fiber was removed using ethyl acetate, followed by surface treatment with sulfuric acid (see Figure S1 for a schematic illustration of fiber fabrication steps). The sulfuric acid treatment effectively removes any organic contaminants from the fiber surface, ensuring that the subsequent alginate (Alg) coating adheres properly and functions optimally as the humidity-sensitive layer. The surface-treated PMMA fibers were coated with Alg through sequential dip coating with sodium alginate solution and a CaCl_2_ solution (Figure 1). This approach resulted in optical fibers with a low refractive index PMMA core and high refractive index alginate cladding, denoted as Alg@PMMA (Figure 1a, b). The thickness of the alginate cladding was tuned by using different coating cycles (see Experimental Section). The polarizing optical microscopy (Figure S2) and SEM images revealed that the pristine PMMA optical fiber exhibits a clear core-cladding structure and a smooth surface morphology (Figure 2). SEM images displayed the PMMA fiber with a cladding thickness of 10 μm for the original cladding (Figure 2a). The ethyl acetate treatment resulted in the removal of the fluorinated cladding from the fiber (Figure 2b). The cross-sectional views based on the SEM images revealed that alginate coating resulted in fibers with a cladding thickness of 57±13 (Alg@PMMA-60), 180±10 (Alg@PMMA-170), and 330±40 μm (Alg@PMMA-370), for one, two, and three cycles of treatments, respectively (Figure 2c–f). The SEM images of Alg@PMMA-370 fibers showed an uneven surface with few crystals, presumably due to excess CaCl_2_ resulting from coagulation (Figure 2e).

**Figure 1:**
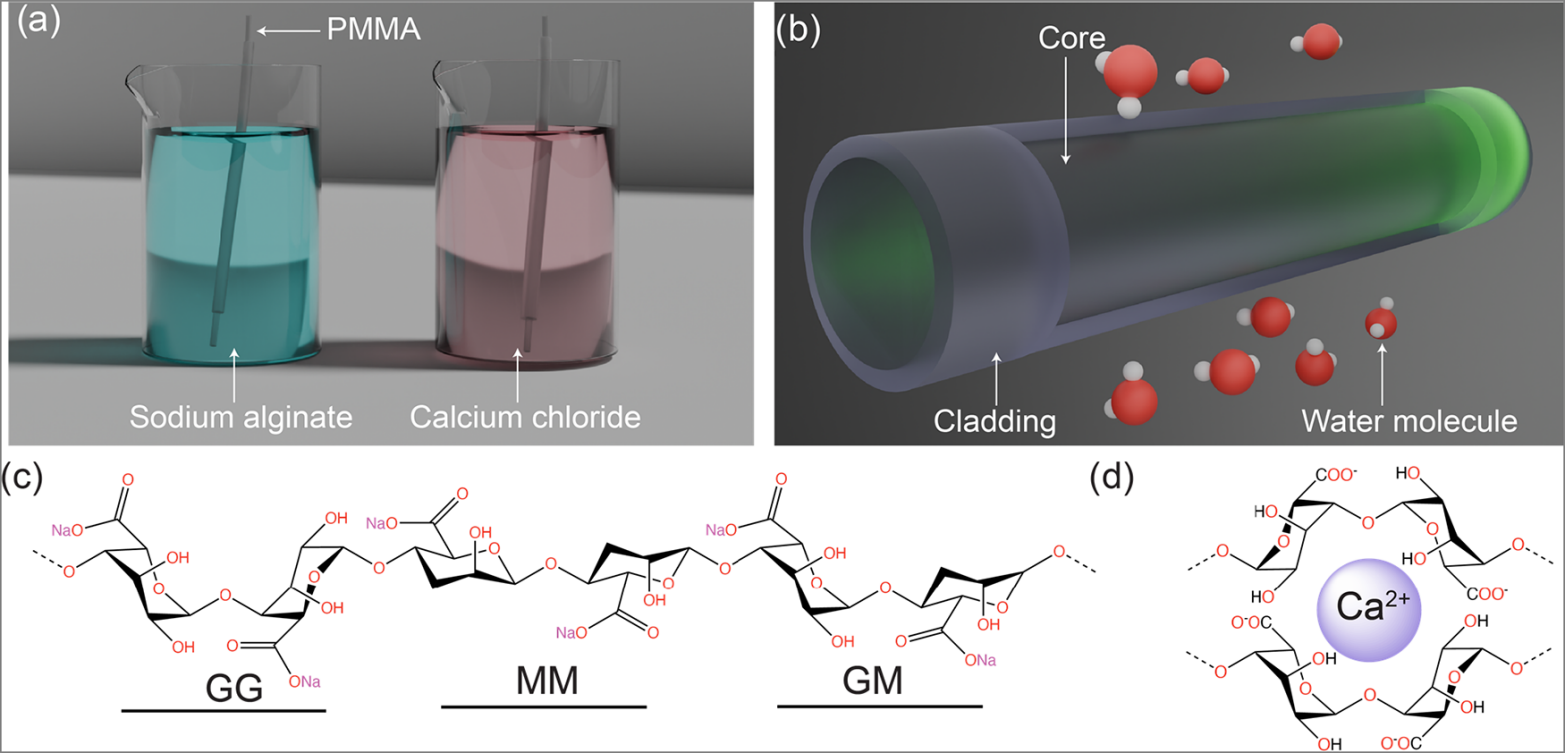
Schematic illustration of fiber fabrication. (a) Illustration of Alg@PMMA fiber fabrication using the dip-coating method. (b) Schematic illustration of the Alg@PMMA optical fiber sensor whose cladding RI is dynamically modulated in the presence of varying humidity. (c) Chemical structure of alginate biopolymer showing β-(1–4)-D-mannuronic acid (M) and α-(1–4)-L-guluronic acid (G) repeat units. (d) Schematic illustration showing metal chelation of alginate.

**Figure 2:**
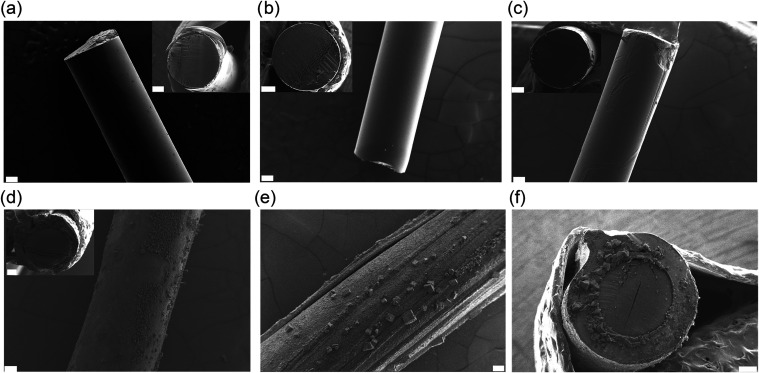
Scanning electron microscopy images of fibers. SEM images showing the surface morphology of the fibers and corresponding cross-sectional view (inset) of the (a) pristine PMMA fiber, (b) cladding-free PMMA fiber, (c) Alg@PMMA-60, (d) Alg@PMMA-170, and (e, f) Alg@PMMA-370. The scale bar corresponds to 200 μm.

The FT-IR spectra of the pristine PMMA fiber displayed the following characteristics: −C–H stretching vibrations of the polymer backbone at 2850–2950 cm^–1^, −C–F stretching from fluorinated cladding at 1284 cm^–1^ and 877 cm^–1^, the −CH_2_ in-plane asymmetric deformation at 1439 cm^–1^, and a small peak at 1159 cm^–1^ corresponding to the −CH_3_ rocking vibration (Figure S3).^79,80^ The FT-IR spectra of ethyl acetate and H_2_SO_4_-treated PMMA fibers showed an intense absorption band at 1724 cm^–1^ attributed to the carbonyl (–C=O) stretching vibration, supporting the removal of the fluorinated cladding.^81^ Furthermore, H_2_SO_4_-treated and Alg@PMMA fibers displayed peaks corresponding to the O–H stretching vibration at 3000–3500 cm^–1^, indicating successful surface activation and alginate coating. Additionally, Alg@PMMA fibers exhibited peaks at 1622 and 1420 cm^–1^, corresponding to the asymmetric and symmetric vibrations of the carboxylate groups, respectively. Finally, a peak at 743 cm^–1^, slightly shifted from the conventional peak at 752 cm^–1^, is assigned to the in-plane –C=O deformation.^80^ The FT-IR spectra suggest successful surface modification of PMMA fibers, which is further corroborated by the optical and electron microscopic images.

### 3.2 COMSOL Simulation of a Model Optical Fiber

The COMSOL simulation was performed using a PMMA fiber with a refractive index of 1.49 as the core and claddings with variable refractive indices ranging from 1.52 to 1.32 (Figure 3).

**Figure 3:**
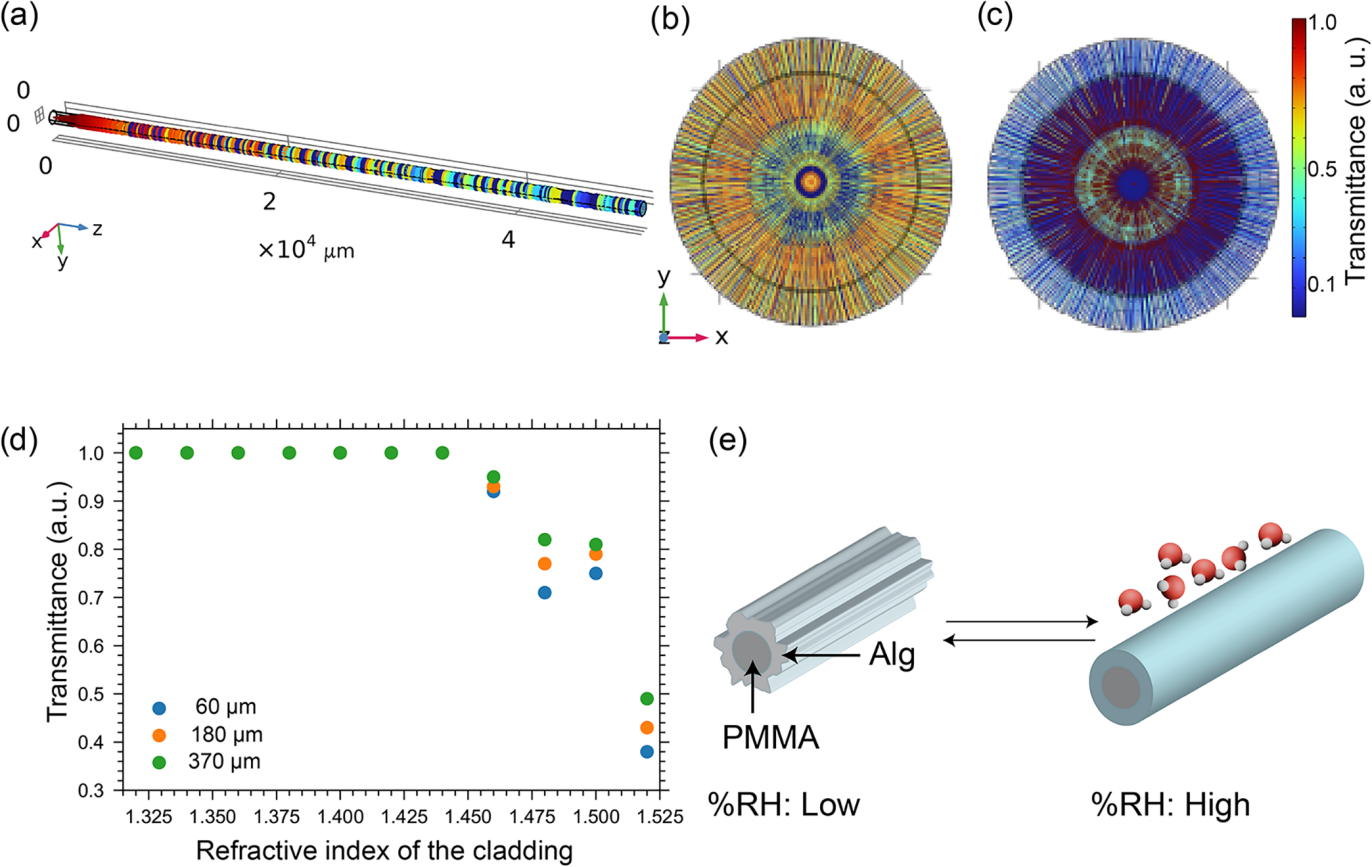
COMSOL Multiphysics simulation of light transmittance. (a) Crystal geometry of a model optical fiber. (b) Cross-sectional view showing the fiber end when the refractive index of the cladding was set to 1.52. (c) Cross-sectional view showing the fiber end when the refractive index of the cladding was set to 1.33. The color of the rays indicates the relative transmittance as they exit the fiber. Higher relative transmittance within the fiber core indicates better optical confinement. (d) Change in the light transmittance as a function of the refractive index of the cladding with different thicknesses. When RI is below 1.46, all Alg@PMMA fibers show improved and similar transmission, resulting in overlapping data. (e) Schematic illustration of dynamically responsive cladding under high and low %RH conditions.

The simulated cladding thickness was used to match our experimental fiber cladding thickness measured using SEM image analysis. The choice of the refractive index of the cladding is based on the experimentally determined refractive index of the alginate film reported in the literature.^82^ Figure 3a shows a simulated light propagation across the geometry for an optical fiber with a low refractive index core and high refractive index cladding. The higher refractive index of the cladding results in high scattering and poor waveguiding. Simulations performed by sequentially reducing the refractive index of the cladding showed an improved transmission and reduced scattering. (Figure 3c). More importantly, a nonlinear change in the light transmission was observed until the refractive index of the cladding was decreased to 1.45 and below which high transmission was observed (Figure 3d). The simulation results suggest the potential of optical fibers for rapid sensing of environmental parameters that dynamically modulate the cladding refractive index.

### 3.3 Evaluation of Humidity Sensing Behavior of Fibers

The hydrophilic nature of alginate enables rapid absorption and desorption of water molecules under varying humidity conditions.^72^ Therefore, we hypothesized that by gradually changing the RH, the refractive index of the alginate cladding in Alg@PMMA fibers can be altered due to the adsorption of water molecules. Accordingly, the light transmission was measured using a ultraviolet–visible (UV–vis) spectrometer (400–1100 nm) by placing the Alg@PMMA fibers inside a custom-designed 3D-printed humidity chamber (Figure 4a).

**Figure 4:**
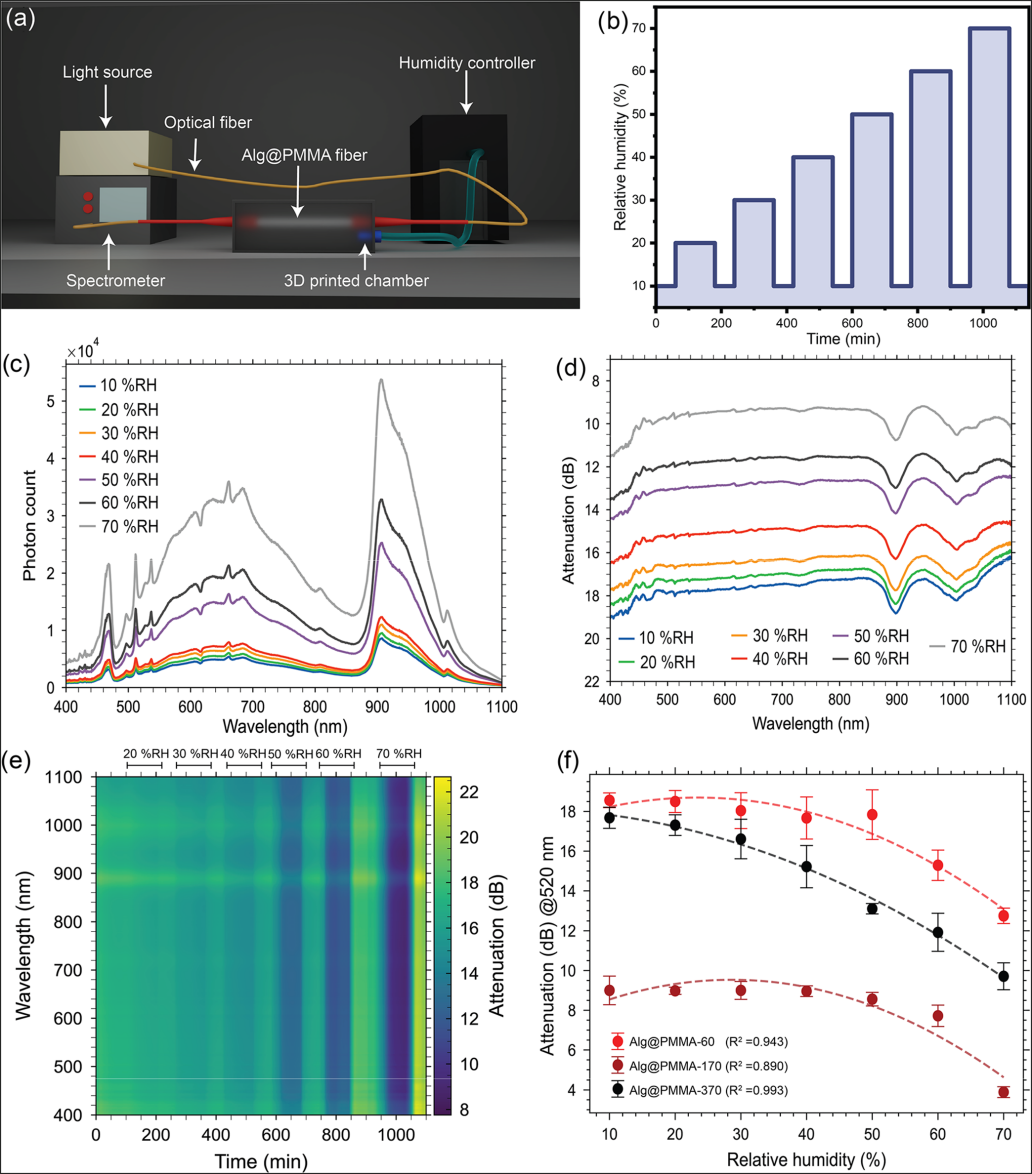
Evaluation of humidity sensing properties of fibers. (a) Schematic illustration of the humidity sensing experimental setup. (b) Illustration of the humidity ramping for controlled relative humidity sensing. (c) Transmission spectra of the Alg@PMMA-370 fiber at different relative humidity levels (10–70% RH). (d) Attenuation spectra of the Alg@PMMA-370 fiber at different %RH levels. (e) Heatmap of attenuation over time across the measured wavelength and varying %RH levels. (f) Attenuation of fibers with different thicknesses of alginate cladding as a function of %RH at 520 nm. The dotted lines show the curves obtained by the polynomial fit.

The humidity was tuned between 10 to 70% RH. The chamber’s environment was initially set to 25 °C and 10% RH and equilibrated for 1 h. Subsequently, the humidity was elevated to 20%RH for two h and then reduced back to 10% RH and equilibrated for 1 h. This cycle with a 10% RH increment step was repeated until a maximum humidity of 70% RH was attained (Figure 4b). The instrument settings dependent photon counts measured under cyclic RH conditions were converted to attenuation values using eq 1 (Figure 4c,d). For Alg@PMMA-370, with increasing RH, transmission improved, as indicated by a decrease in the level of attenuation (Figure 4d). For all Alg@PMMA fibers, the highest photon count was observed at 70% RH. More importantly, a rapid change in attenuation was observed above 40% RH for Alg@PMMA-370 fiber. On the other hand, in the case of Alg@PMMA-60 and Alg@PMMA-170, a rapid change in attenuation was observed above 50% RH (Figure S4). Attenuation spectra show that Alg@PMMA-370 fibers show a linear response to changes in %RH, with a difference of nearly 8 dB between 10 and 70% RH (Figure 4d).

More importantly, the attenuation remains similar until 50% RH for Alg@PMMA-60 and Alg@PMMA-170 fibers, with a rapid decrease in attenuation beyond that RH, resulting in an overall change of 4 dB (Figure 4f). This suggests that the cladding thickness plays a major role in humidity sensing and response. At lower humidity, significant losses can occur due to scattering losses through the cladding and core-clad interface. While the cladding has a higher RI than the core, losses remain high due to light coupling into loss modes in the cladding layer. Initially, the absorbed moisture diffuses toward the core–cladding interface, reducing the refractive index contrast between the core and the Alg cladding. At this stage, effective waveguiding does not occur. However, once the moisture absorption lowers the RI of the Alg cladding below that of the core, a functional core–cladding structure is established, enabling waveguiding and significantly decreasing optical losses. As additional moisture is absorbed, the refractive index contrast continues to increase, broadening the total internal reflection (TIR) angle and enhancing the light coupling into guided modes. This supports the hypothesis that upon increasing the RH, the cladding refractive index decreases, promoting the TIR with improved transmission. The dynamic response of the sensor was measured by varying the %RH as presented in Figure 4e, where brighter bands and an increase in attenuation were observed when the humidity was reduced to 10% RH. The light transmission (dB) at 520 nm under varying humidity for Alg@PMMA fibers with polynomial fit indicates a strong correlation (*R* ^2^ = 0.94, 0.89, 0.99, respectively for Alg@PMMA-60, Alg@PMMA-170, and Alg@PMMA-370), confirming the robustness of the trend (Figure 4f). The coefficients of these curves obtained using second-order polynomials indicate that the optical losses decrease with increasing humidity. The humidity response is more pronounced as the thickness of the coating increases. The Alg@PMMA-370 fiber exhibited the highest transmission improvement of 9.4 dB at 70% RH and showed the highest correlation (*R* ^2^ = 0.99). With the increase in RH, water molecules are absorbed into the Alg@PMMA fiber, which subsequently reduces the refractive index of the cladding, resulting in a low optical loss (Figure 4f). This effect is hypothesized to be more pronounced in thicker coatings due to their increased capacity for the absorption of water molecules. The thicker coating would provide a more uniform layer and a continuous interface, reducing scattering losses and improving light transmission. This effect was observed over all of the coating thicknesses, with the highest light transmittance at 70% RH for both Alg@PMMA-60 and Alg@PMMA-170 fibers (Figure S5a, c). This is also evident in the reduced attenuation by increasing the RH, where the transmission improves drastically at higher RH (Figure S5c, d).

To further confirm the effect of alginate cladding, similar experiments were performed using commercial, ethyl acetate-treated, cladding-free, and H_2_SO_4_-treated PMMA fibers (Figure S6). For commercial PMMA fibers, minimal improvements in transmission at higher ambient humidity are observed as moisture condenses on the fibers, acting as a temporary cladding layer (Figure S7). The 1 dB improved transmission occurring only after 60 to 70% RH step is an order of magnitude lower improvement than with Alg@PMMA fibers (Figure S7a, b). On the other hand, upon removing the cladding, a slight increase in attenuation was observed, which could be due to the uneven surface of the fiber enabling increased light scattering (Figure S7c). As a result, pronounced changes in attenuation were not observed (Figure S7d). The sulfuric acid treatment activates the fiber surface, and the presence of hydrophilic carboxylic acid moieties enables the condensation of water molecules on the surface, resulting in temporary cladding (Figure S7e). Water has a refractive index of 1.33,^81^ which is lower than the core’s refractive index, improving TIR, thereby reducing the attenuation by 2 dB at higher RH. A decrease in attenuation was observed (4.5 to 3.5 dB) for the H_2_SO_4_-treated fiber (Figure S7f). This emphasizes the importance of removing the cladding and further surface treatment for humidity sensing applications. Increasing the RH produced limited improvements in transmission for all stages of PMMA preparation, with an overall attenuation change of 0.5–1.0 dB (Figure S8).

### 3.4 Sensor Integration for Breath Humidity Monitoring

Fluctuations in breath humidity, characterized by response and recovery periods of approximately 3 to 5 s, occur synchronously with respiration. These variations are crucial in breath monitoring, particularly for providing data on the respiratory rate, nasal versus oral exhalation, and breathing patterns. To test the feasibility of our fiber sensors for breath humidity monitoring, a proof-of-concept miniaturized device was fabricated (see Experimental Section, Figure S9). The prototype consists of a light-emitting diode (LED) as the light source and a photodiode as the detector, translating changes in RH to changes in voltage. This photodiode was further connected to a transimpedance amplifier circuit, which was in turn connected to an Arduino Nano microcontroller for data collection (Figure S9). First, the breathing response experiment was performed for all three Alg@PMMA fibers. When exhalation was performed on the Alg@PMMA-60 fiber, an instantaneous response was observed (Figure S10a). On the other hand, Alg@PMMA-170 and Alg@PMMA-370 fibers required 125 and 350 s, respectively, to show a significant rise in the voltage (Figure S10b, c). This is presumably due to the accumulation of water molecules on the surface of the thick cladding, requiring more time for water molecules to percolate the entire thickness and reach the cladding-core interface, where the effects of water in the Alg polymer matrix affect the transmitted light.

To identify the optimal light source, the performance was evaluated by using different light-emitting diodes (LEDs) as the light source, and the voltage output was monitored. With a clear lens red LED operating at 640 nm wavelength, there was a lower output voltage of 0.5 V at ambient humidity (24% RH), but oral and nasal exhalation was prominent (Figure S11a). However, with a yellow-colored LED operating at a 580 nm wavelength, there was a further reduction in the output voltage to 0.1 V, and the oral and nasal exhalation responses were poor (Figure S11b). Among all fibers, Alg@PMMA-60 showed a rapid response compared to other fibers. Therefore, we calibrated it under controlled humidity conditions from 10–80% RH and monitored the voltage change. For calibration of the sensor, the fiber was placed inside a 3D-printed chamber and humidity was controlled using a Linkam RH95 humidity controller. The humidity was ramped from 10 to 80% RH, with each step of 10% RH maintained for 1 h. Upon increasing the RH, a significant increase in the voltage from 0.4 to 1.2 V was observed (Figure S12a). The adsorption curve remains relatively stable at lower humidity levels but exhibits a noticeable increase beyond 50% RH. This suggests a threshold RH at which the sensor significantly responds. A similar trend was observed for the desorption curve but it exhibits a hysteresis effect. This suggests that the material may retain a certain amount of moisture, especially at 40% RH, resulting in slow desorption. The response followed a similar exponential rise in response as the humidity increased, and a second-order polynomial equation was fitted with *R* ^2^ of 0.87 (Figure S12b). A linear term originates from the direct refractive index shifts associated with changes in humidity, and the quadratic term indicates plausible multilayer adsorption.

To evaluate the feasibility of the device for real-time breath humidity monitoring, we used the Alg@PMMA-60 fiber placed across the LED and photodiode (Figure 5a). The measurement was calibrated based on the output voltage with a second-order polynomial equation, presented across different breathing modes of volunteer#1: shallow, nasal breathing, oral deep breathing, and shallow oral breathing. A significant rise in the signal was observed upon transition from shallow to deep breathing. This occurs as the oral breath contains higher moisture content compared to the nasal breath. As the exhalation ceases, the humidity level stabilizes back to the ambient condition and takes 150 s for the total desorption to occur. Magnified regions of shallow and deep exhalations (Figure 5b, c) of volunteer #1 reveal the sensor’s rapid response and recovery time of 1.37 and 1.4 s, respectively.

**Figure 5:**
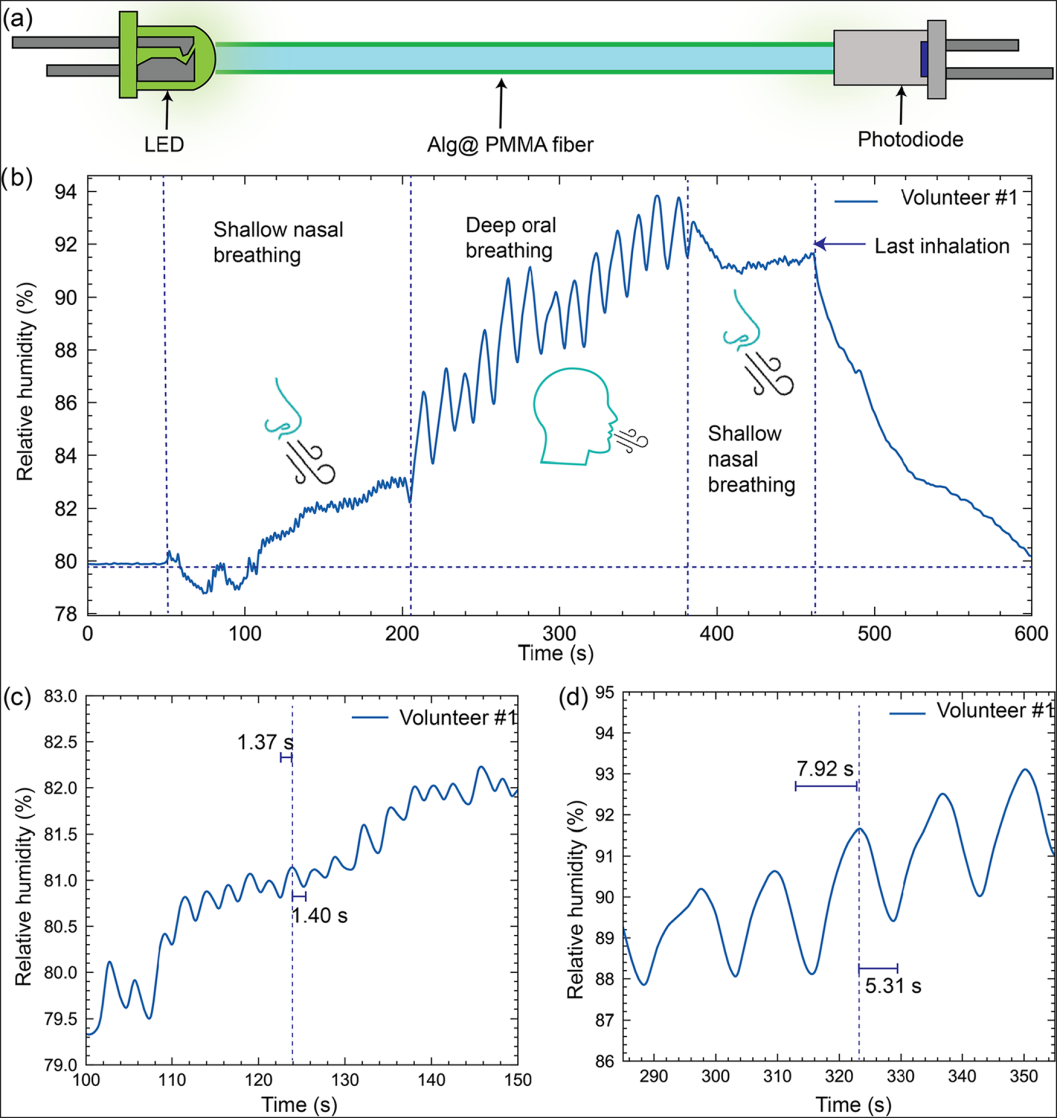
Breath humidity monitoring and differentiation using a miniaturized sensor platform. (a) Schematic illustration of the breath humidity sensing setup and monitoring at 520 nm through the Alg@PMMA-60 fiber. (b) Change in the humidity upon breathing by volunteer no. 1, showing transitions between shallow nasal and oral breathing, back to shallow nasal breathing, with the last inhalation marked. (c, d) Magnified regions of humidity variations corresponding to different breathing phases of volunteer #1, highlighting the characteristic period between breaths.

The measured humidity change for shallow nasal breathing corresponds to a lower peak, while deep oral exhalation results in a significantly higher peak. This increase in amplitude can be attributed to the greater volume of air expelled during deep breathing, resulting in increased moisture deposition on the sensor’s surface. The exhalation cycle during the deep oral breathing was 7.9 s, whereas the inhalation cycle was 5.92 s, indicating longer breathing cycles. These observations highlight the sensor’s ability to resolve temporal and respiratory patterns based on humidity fluctuations, providing vital insights into breath intensity and depth. Additionally, the sensor does not exhibit saturation, as the signal amplitude continues to rise even with prolonged exhalation. This demonstrates the sensor’s robustness in handling extended high-humidity cycling, making it suitable for long-term respiratory monitoring applications. The dynamic variations between oral and nasal breathing of volunteer no. 2 are shown in Figure 6a. The initial oral exhalation results in a higher humidity response, as marked in the oral breathing region, which decreases upon switching to nasal breathing. Systematic analysis shows the response and recovery times of 2.63 and 2.69 s, respectively, for oral and nasal exhalation, with a peak humidity response corresponding to the increased moisture content in the exhaled breath (Figure 6b, c). On the other hand, nasal exhalation exhibited a slightly more extended inhalation and exhalation duration, marked by a response and recovery of 2.7 and 2.71 s, respectively, with a lower amplitude. While several fiber optic-based sensors designed using highly sophisticated fabrication and coating are known in the literature, the response time and recovery time of our sensor are equal to or better than several single-mode glass optical fiber-based humidity sensors (see Table S1 for comparison). Our findings validate the sensor’s capability to measure and distinguish individual respiratory patterns through precise temporal and amplitude-based humidity analysis. We further evaluated the comprehensive cost breakdown of the electronic components used in the proof-of-concept device (Table S2). The overall cost of the proof-of-concept device ranges from USD 36 to 59. The prices provided reflect the potential variation between the suppliers and the quantity of each specific component. This cost can be further reduced through further miniaturization by utilizing surface-mount components and purchasing in bulk quantities.

**Figure 6:**
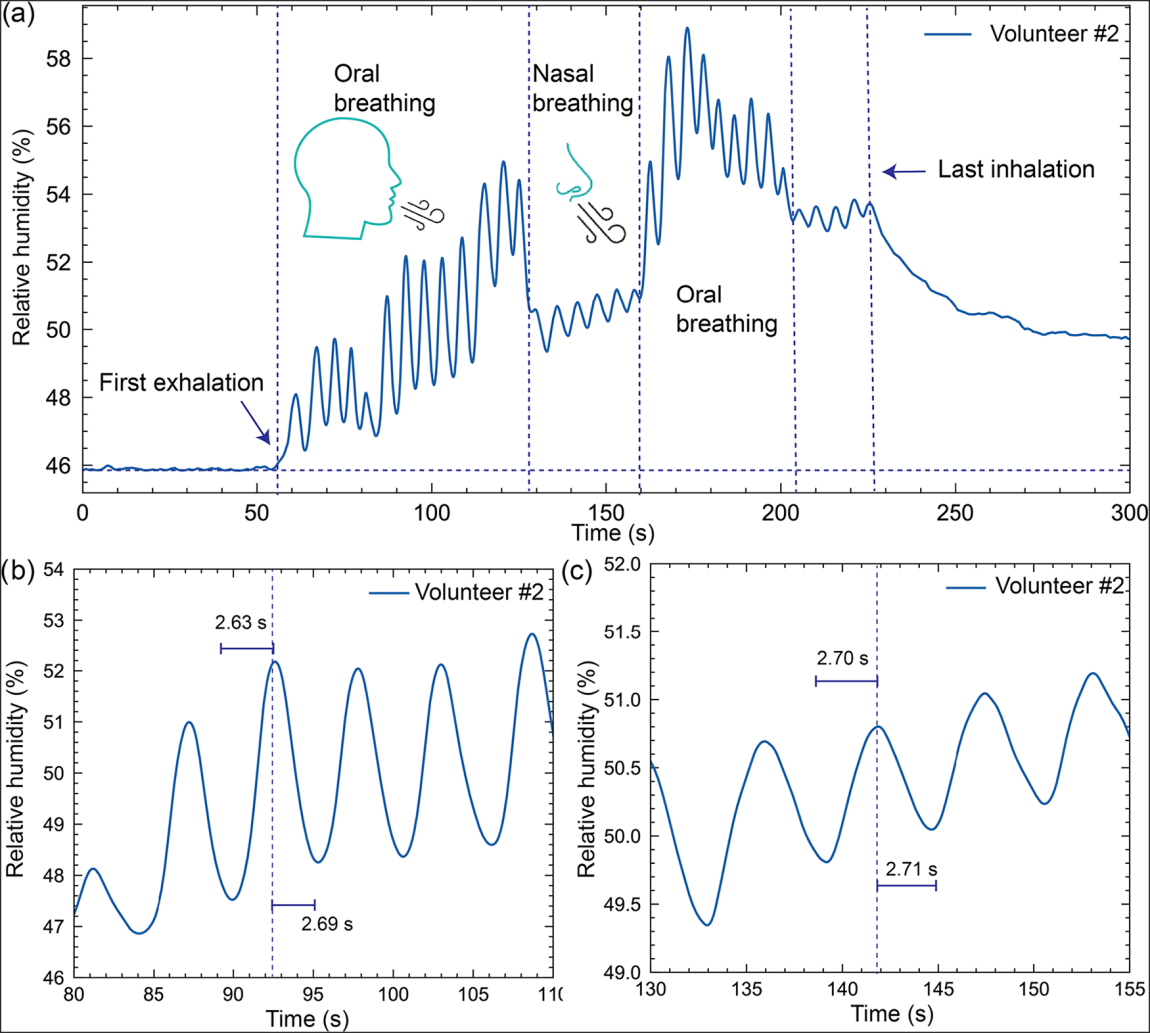
Monitoring of periodic breath patterns. (a) Variation of relative humidity upon breathing by variation for volunteer #2 on the Alg@PMMA-60 fiber, depicting changes during oral and nasal breathing phases. (b, c) Expanded region showing %RH variations for volunteer #2, showing periodic breathing patterns with measured time intervals marked operated using a green LED working at 520 nm.

Since the highest transmission was observed in the NIR region from 900 to 1000 nm (Figure 4b–d), we replaced the green LED with LED930L (operating at a wavelength of 930 nm) and used a glass lens with a spectral range of 830–980 nm with a peak maximum at 930 nm. The nasal and oral breathing of volunteer no. 2 was performed using the Alg@PMMA-60 fiber (Figure S13). Nasal exhalation was performed between 30 to 100 s, with a peak amplitude of 0.05 s with an average period of 5 s for inhalation and exhalation. Upon oral exhalation, the peak amplitude was 0.18 V with an average time of 6 s. A marked region was presented when the volunteer was asked to hold their breath, resulting in humidity desorption from the fiber’s surface. After 30 s of withholding, the volunteer was asked to exhale through their mouth, which increased the output voltage as soon as the humidity from the orally exhaled breath affected the refractive index of the cladding. To further evaluate the performance of the device at 930 nm LED, volunteer no. 2 orally exhaled on the sensor for 10 min (Figure S13). There was an overall increase in the voltage readout, which increases with continuous breathing over the sensor. The saturation of output voltage was not observed within 10 min of constant breathing, suggesting the sensor could be used for long-term breath monitoring. The breath cycle takes 10 s for volunteer #2 with a peak amplitude of 0.12 s. Furthermore, the amplitude of different oral and nasal breathing peaks was resolved more accurately (35%) than that of the green LED. However, in terms of cost, LED930L is 100 times more expensive than the green LED. Alternative sources that mass-produce green LEDs could provide more affordable options for 930 nm LEDs. Fabrication of the proof-of-concept device using this affordable LED will be pursued in the future.

## 4 Conclusions

Humidity is one of the critical environmental parameters affecting human health, agriculture, food storage and transportation, building maintenance, and electronics manufacturing. Even minor fluctuations in humidity levels can affect respiratory functions, safety, operational efficiency, and material integrity. More importantly, the breath humidity is an essential indicator of respiratory function and overall well-being. Real-time, accurate, and noninvasive monitoring of breath humidity enables the detection of abnormal breath patterns associated with conditions such as asthma, COPD, and lung functions, which is valuable for timely clinical intervention. Here, we fabricated a sensor for real-time breath humidity monitoring utilizing an alginate biopolymer as the cladding with a dynamically responsive refractive index on a PMMA optical fiber. The optical fiber was readily integrated into a miniaturized sensor platform using a cost-effective LED light source and a photodiode. Our proof-of-concept device demonstrates the ability to dynamically detect nasal and oral breathing based on the amplitude and time of the peak of chrono-voltametric spectra. The sensor exhibits a rapid response and recovery times of 1.37 and 1.40 s, respectively, for shallow oral breathing. Furthermore, the exhalation and inhalation cycles for deep oral breathing were found to be 7.92 and 5.31 s, respectively. Apart from distinguishing different oral breath patterns, the sensor readily distinguishes oral and nasal breathing. The device does not exhibit saturation, as demonstrated by using continuous breathing for approximately 10 min. It demonstrates the sensor’s ability and robustness in performing under high humidity and cyclic humidity changing conditions. Such devices are suitable for long-term respiratory monitoring. The alginate cladding may be susceptible to degradation over time due to its ability to absorb moisture from the environment. This hydrophilic property can result in increased water absorption, which may alter the mechanical integrity of the alginate material and induce breakdown, particularly if exposed to high humidity or direct water contact for an extended duration. We stored the samples at ambient conditions (22 °C and <45% RH) for 300 days with no significant variation in the performance. However, the Alg@PMMA-60 samples after 30 cycles of switching from 70 to 10% RH resulted in visible cracks on the coating. This was not observed for the other thicknesses. However, during breathing experiments, the Alg@PMMA-60 sample did not degrade even after 100 breathing cycles. Although the fiber is sensitive to breathing, showing a rapid response and recovery for a complete desorption of the fiber, it takes 120 s. False positive results in miniaturized sensors, i.e., signal detection without breathing onto the fiber under controlled conditions, are rather unlikely. However, if the fiber is saturated with moisture, one might see a weak signal resulting in false negative cases. Furthermore, if an individual has a dry mouth or low moisture content in exhaled breath, the detection can be limited. Low signal detection may arise from a volunteer breathing with a blocked nose due to conditions such as cold or the flu, limiting nasal breathing detection. Overall, the operation at room temperature and relying solely on refractive index changes without involving chemical reactions, the sensor offers low power consumption, excellent reversibility, and long-term durability. Additionally, the use of alginate ensures biocompatibility, eco-friendliness and ease of fabrication, making the sensor platform a promising candidate for noninvasive, long-term respiration monitoring, and other humidity sensing applications.

## Supplementary Material



## Notes

Schematic of the sensor fabrication process; optical microscopy images of the fibers; FT-IR spectra; light transmission as a function of alginate coating thickness; light transmission under varying humidity for surface-treated PMMA fiber; attenuation as a function of relative humidity of PMMA fibers before and after surface treatment; schematic representation of the electronic circuit; cost-estimates of materials; chrono-voltammetry spectra of Alg@PMMA fibers; chrono-voltammetry of breathing response using different LEDs; calibration curves of %RH and voltage; comparison of responses with published literature, and chrono-voltametric response of breathing at 930 mm LED (PDF)
