# Supplementary material for: Optical Fiber Sensor with Dynamically Responsive Cladding for Real-Time Breath Pattern Monitoring
Source: ACS Omega. 2025 Sep 19;10(38):44528–40. doi: 10.1021/acsomega.5c06598 (PMC12489730; doi:10.1021/acsomega.5c06598)
Supplement: Supplementary file 1 [file ao5c06598_si_001.pdf]

Supporting Information

Optical Fiber Sensor with Dynamically Responsive Cladding for Real-Time Breath Pattern Monitoring

Pillalamarri Srikrishnarka,[a] Jani Patrakka,[a] Zhipei Sun,[b] and Nonappa [a,*]

[a]Faculty of Engineering and Natural Sciences, Tampere University, Korkeakoulunkatu 6, FI-33720, Tampere, Finland.

[b]Department of Electronics and Nanoengineering, Aalto University, Maarintie 13, Espoo, 02150 Finland.

Table of Contents

Figure S1: Schematic illustration of surface treatment of PMMA optical fibers……………………S2

Figure S2: Polarizing optical microscopy (POM) imaging…………………………………….....S3

Figure S3: FT-IR spectroscopy of fibers…………………………………………………...S4

Figure S4: Flowchart for measuring attenuation and relative humidity sensitivity of Alg@PMMA...S5

Figure S5: Transmission spectra and attenuation spectra of Alg@PMMA fibers with varying %RH..S6

Figure S6: Transmission and attenuation spectra of PMMA optical fibers…………………………..S7

Figure S7: Transmission spectra and attenuation spectra of PMMA fibers with varying %RH………S8

Figure S8: Attenuation as a function of %RH of PMMA optical fibers………………………………S9

Figure S9: Schematics showing electronic circuit of sensor prototype………………………………S10

Figure S10. Choronovoltametry spectra of Alg@PMMA fibers……………………………………...S11

Figure S11. Choronovoltametry spectra Alg@PMMA-60 using 640 nm LED 580 nm LEDs……....S12

Figure S12. Humidity response and sensor calibration for Alg@PMMA-60...................................S13

Table S1. Comparison of the properties of different fiber optic-based humidity sensors…S9

Table S2. Cost-estimate of components and materials……………………………………………….S14

Figure S13. Chronovoltametric response of Alg@PMMA-60 breathing under 930 nm LED……….S15

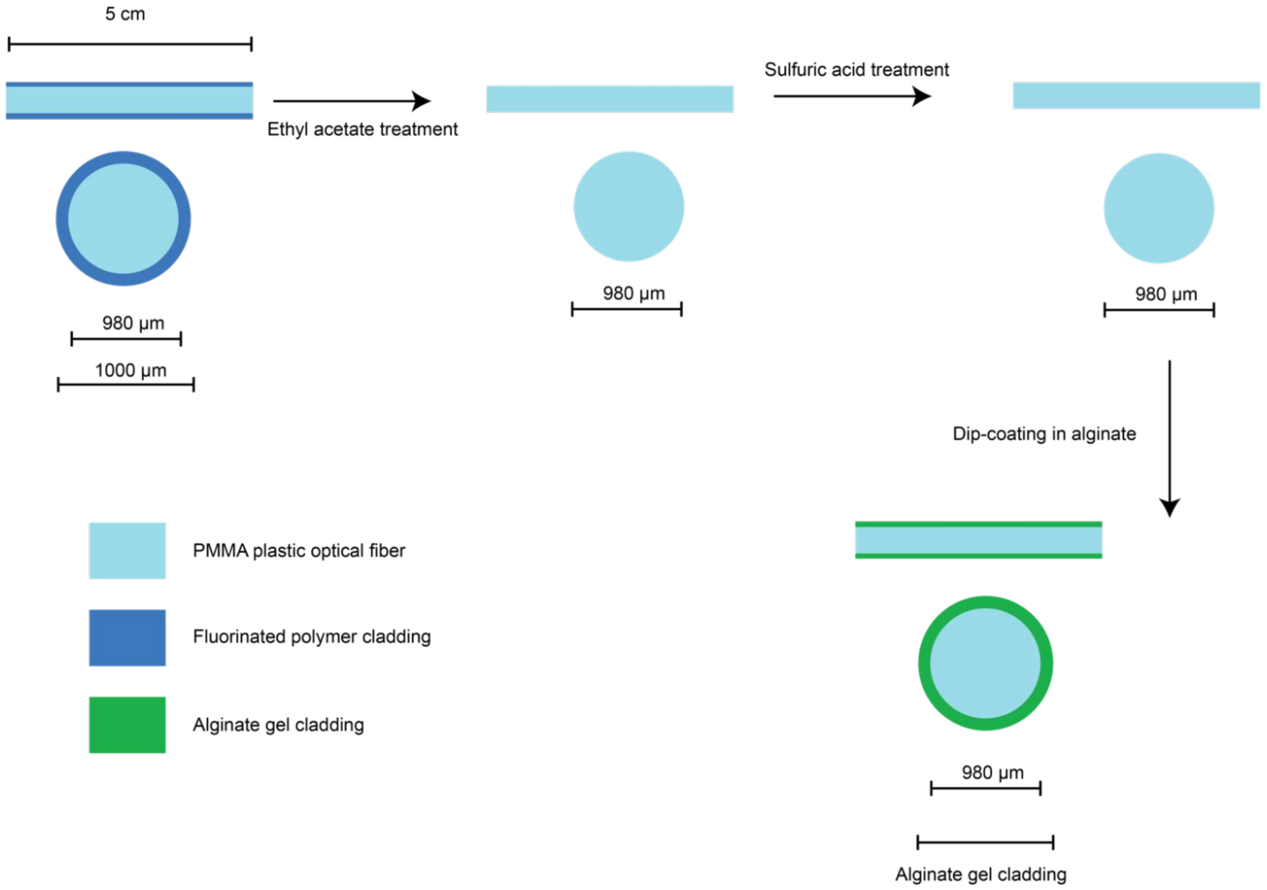

Figure S1. Schematic illustration of surface treatment of PMMA fiber and coating of alginate biopolymer to obtain Alg@PMMA fibers.

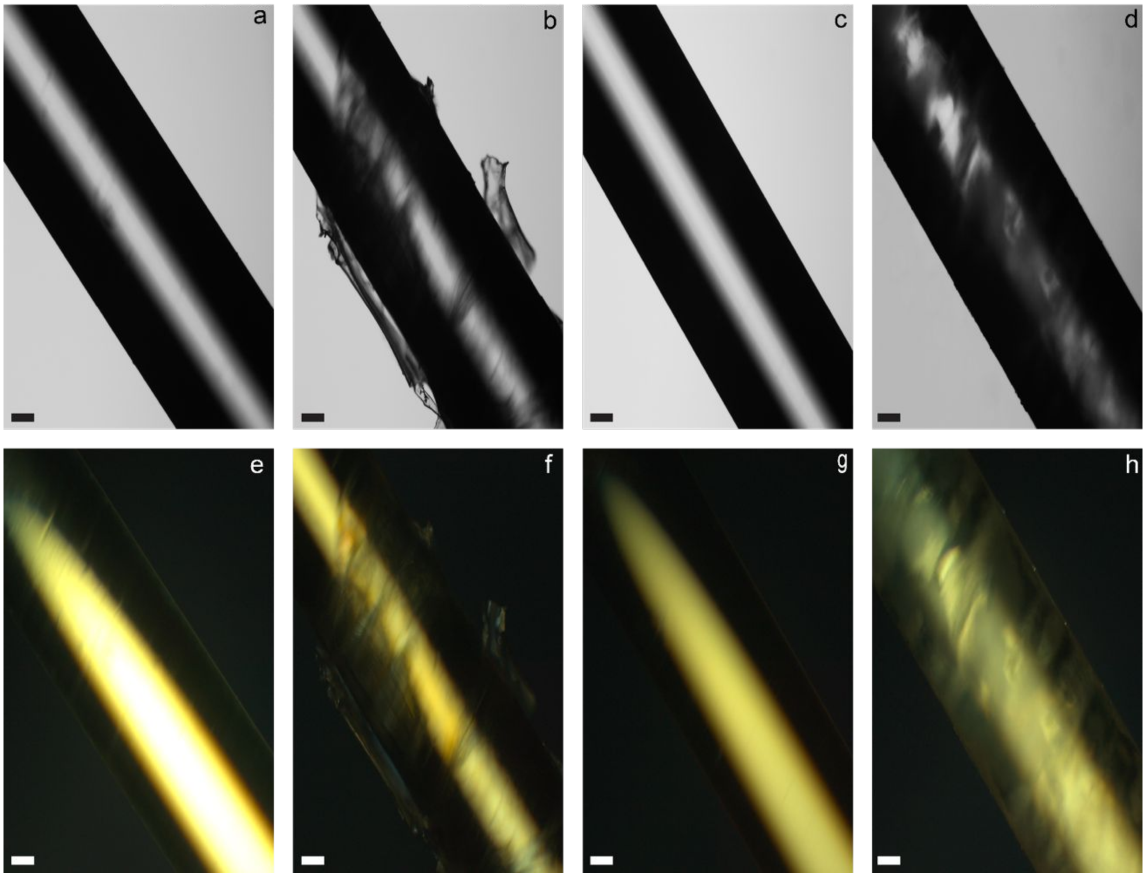

Figure S2. Polarizing optical microscopy (POM) imaging. Bright field POM images of (a) pristine PMMA optical fiber, (b) Alg@PMMA-60, (c) Alg@PMMA-170, and (d) Alg@PMMA-370. (e-h) Shows corresponding dark field POM images. The scale bar corresponds to 200 μm.

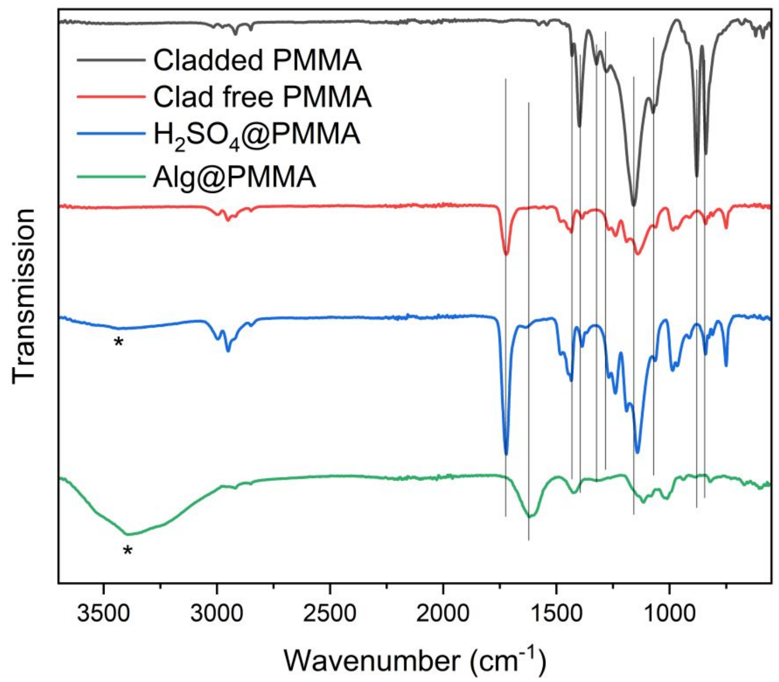

Figure S3. FT-IR spectra of PMMA optical fiber before and after surface treatment**.**

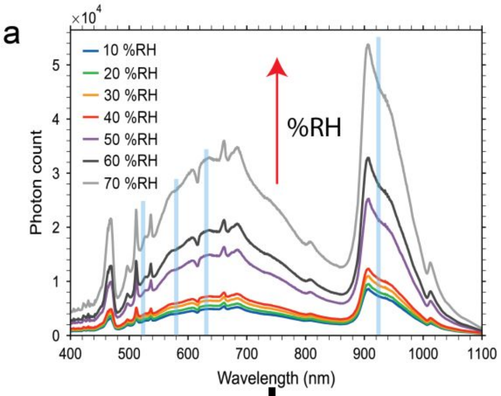

Optical signal transmitted in the PMMA optical fiber was measured in photon counts per incident wavelength. Integration time is an important instrumental parameter that influences the output photon count. Further, the recorded photon counts were converted into optical looses (dB) by comparing with the baseline photon counts having no optical fiber coupled. Transmission looses were calculated based on Eq 1:

$$Attenuation\ (dB) = 10 * \log_{10} \frac{I_s(\lambda)}{I_B(\lambda) * \Delta t} \qquad (1)$$

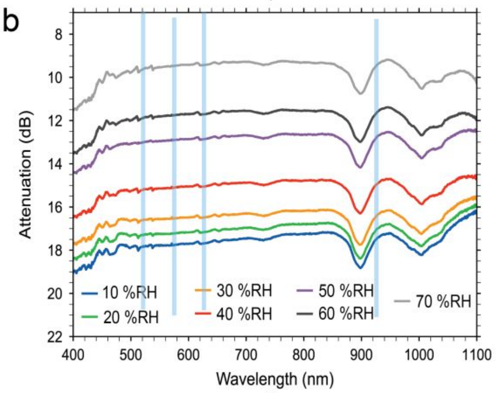

The optical losses or attenuation at a particular wavelength was measured by taking the ratio of photon count from the sample (I_s) to the baseline photon counts without the sample (I_B). Instances where the integration time differs for the sample and baseline, then the baseline photon count was multiplied with the ratio of sample and baseline integration times $\Delta t = t_S/t_B$.

The optical transmission T at select few wavelengths (520, 580, 640 and 930 nm) are plotted across different relative humidity levels and a linear/polynomial fit was performed to obtain the humidity sensitivity at particular wavelenghts as a fitting gradient. Samples which showed poor (R^2) where fitted using polyomial function.

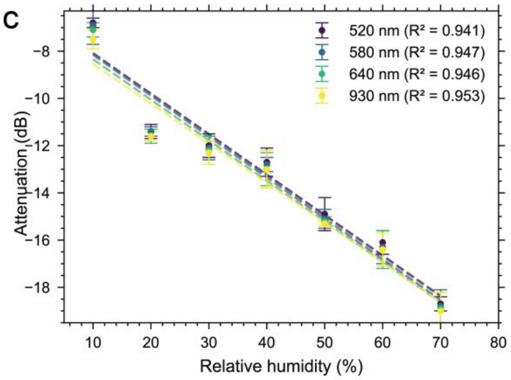

Alg@PMMA-370

520 nm: -0.18x - 6.37
580 nm: -0.17x - 6.42
640 nm: -0.17x - 6.62
930 nm: -0.16x - 6.85

Figure S4: Flowchart for measuring attenuation and relative humidity sensitivity. (a-c) Shows the workflow of the conversion of photon counts into attenuation across 400-1100 nm wavelengths upon changing the humidity. Here Alg@PMMA-370 is used as an example.

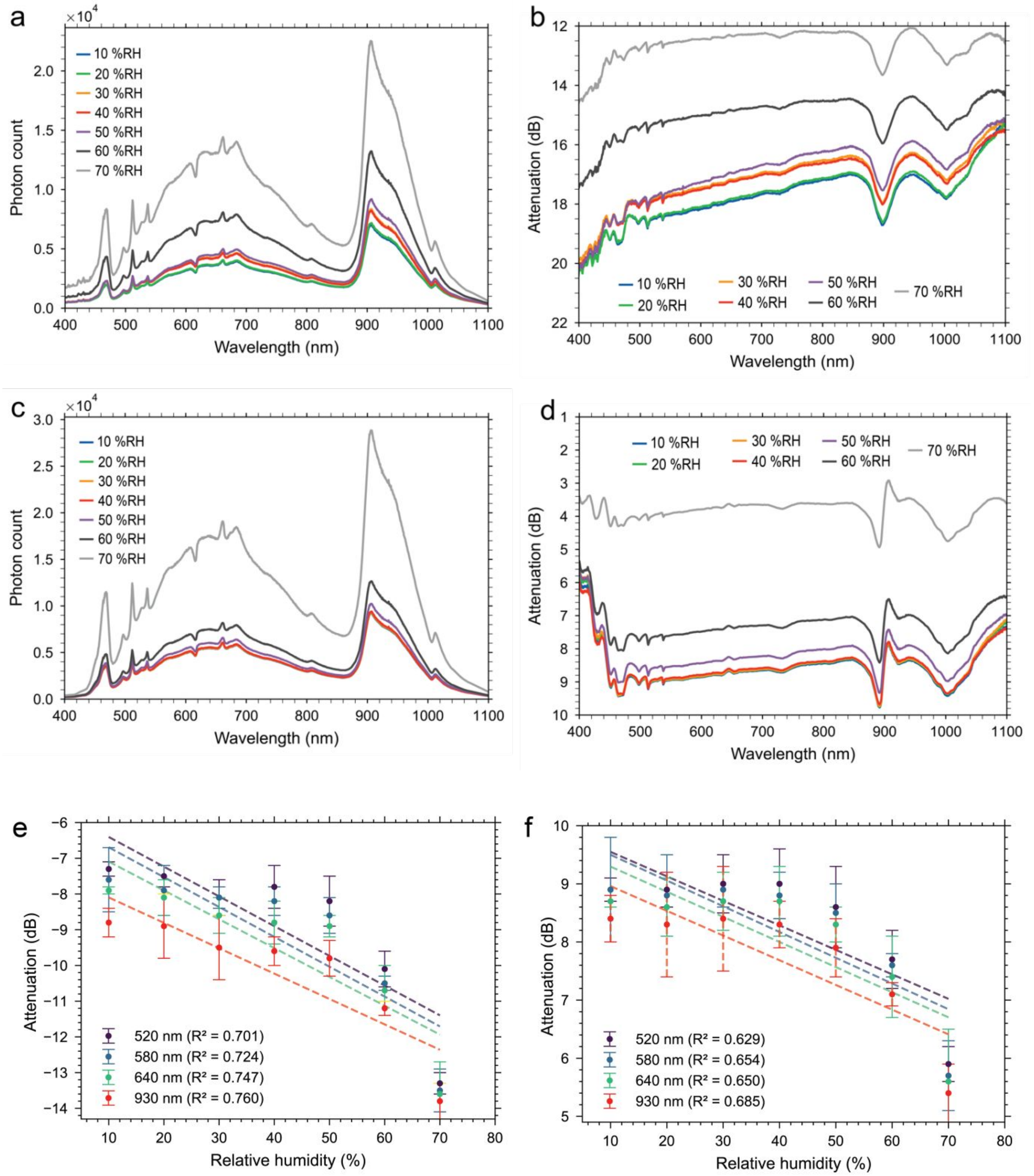

Figure S5. Transmission spectra and attenuation measurements across 400-1100 nm with varying %RH. (a) Fiber transmission spectra and of Alg@PMMA-60. (b) Attenuation spectra and of Alg@PMMA-60. (c) Fiber transmission spectra and of Alg@PMMA-170. (d) Attenuation spectra and of Alg@PMMA-170. (e and f) Attenuation as a function of %RH in selected wavelengths of Alg@PMMA-60 and Alg@PMMA-170, respectively.

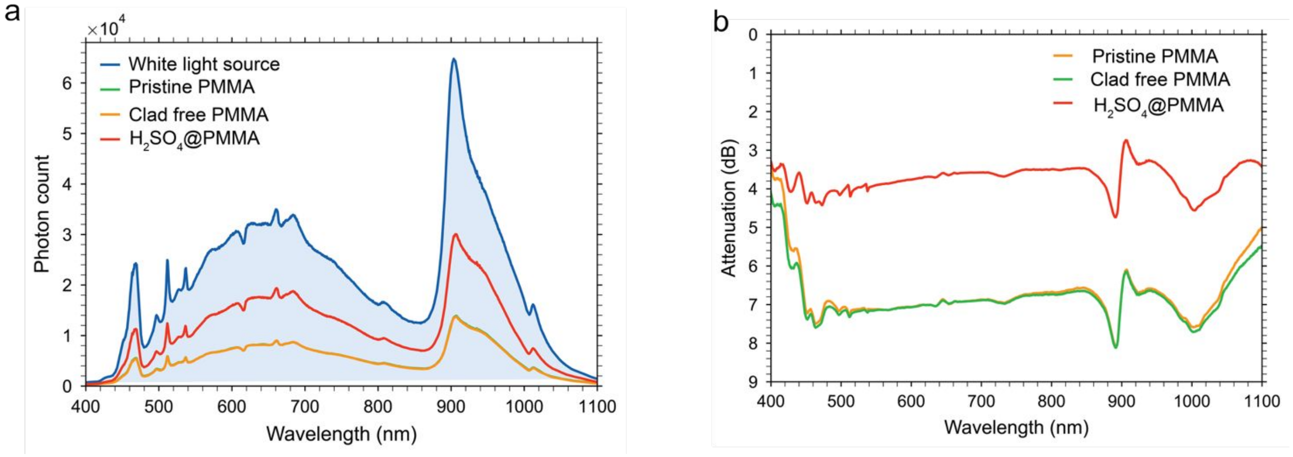

Figure S6. Transmission spectra across 400-1100 nm using white light source. (a) Transmission spectra of pristine, and surface treated PMMA fiber using white light source. (b) Calculated attenuated spectra of the fibers.

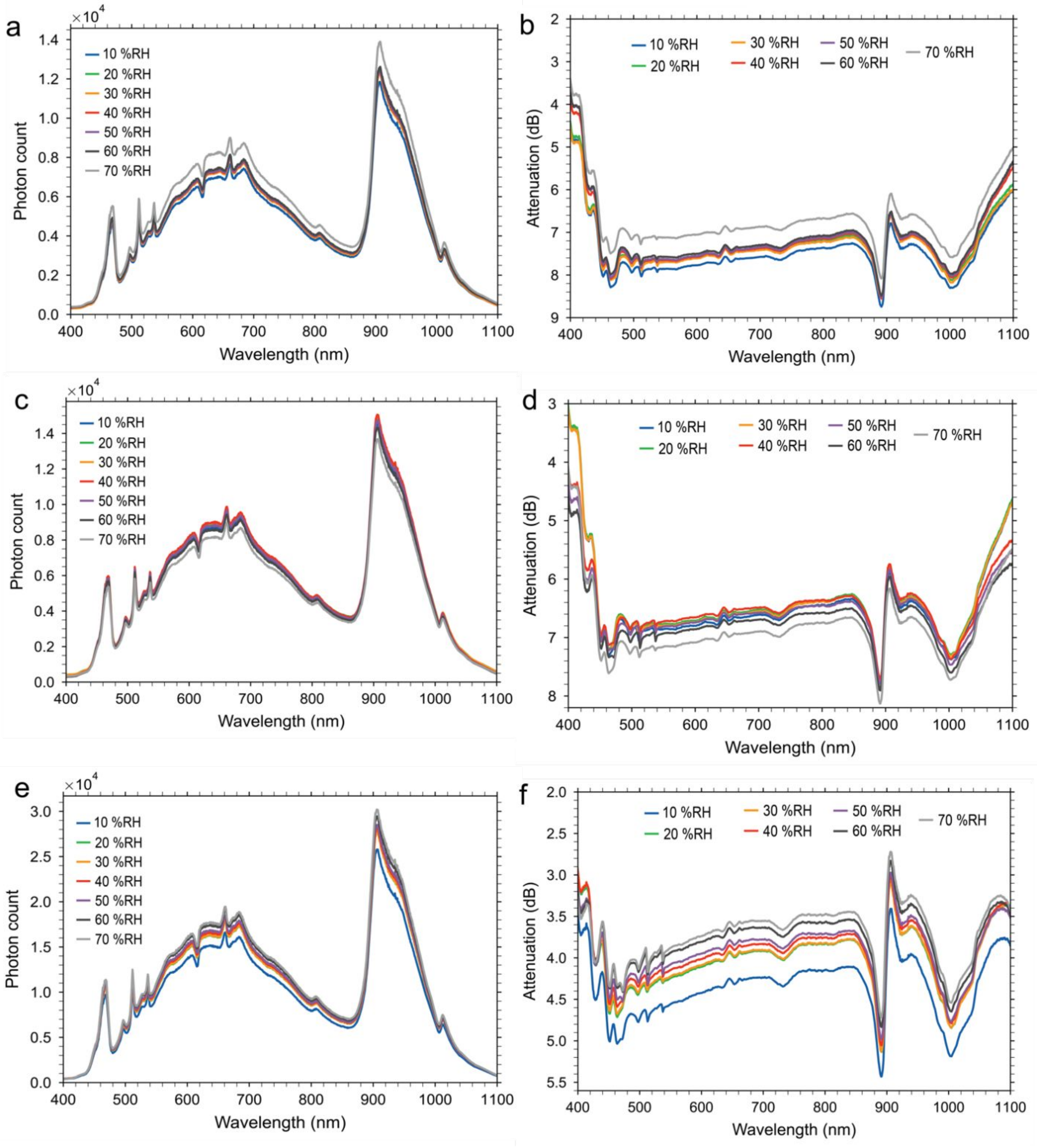

Figure S7. Transmission spectra and attenuation spectra of PMMA fibers across 400-1100 nm. (a and b) Transmission and attenuation spectra of PMMA optical fiber. (c and d) Transmission and attenuation spectra of cladding-free PMMA optical fiber. (e and f) Transmission and attenuation spectra of H_2SO_4-treated PMMA optical fiber.

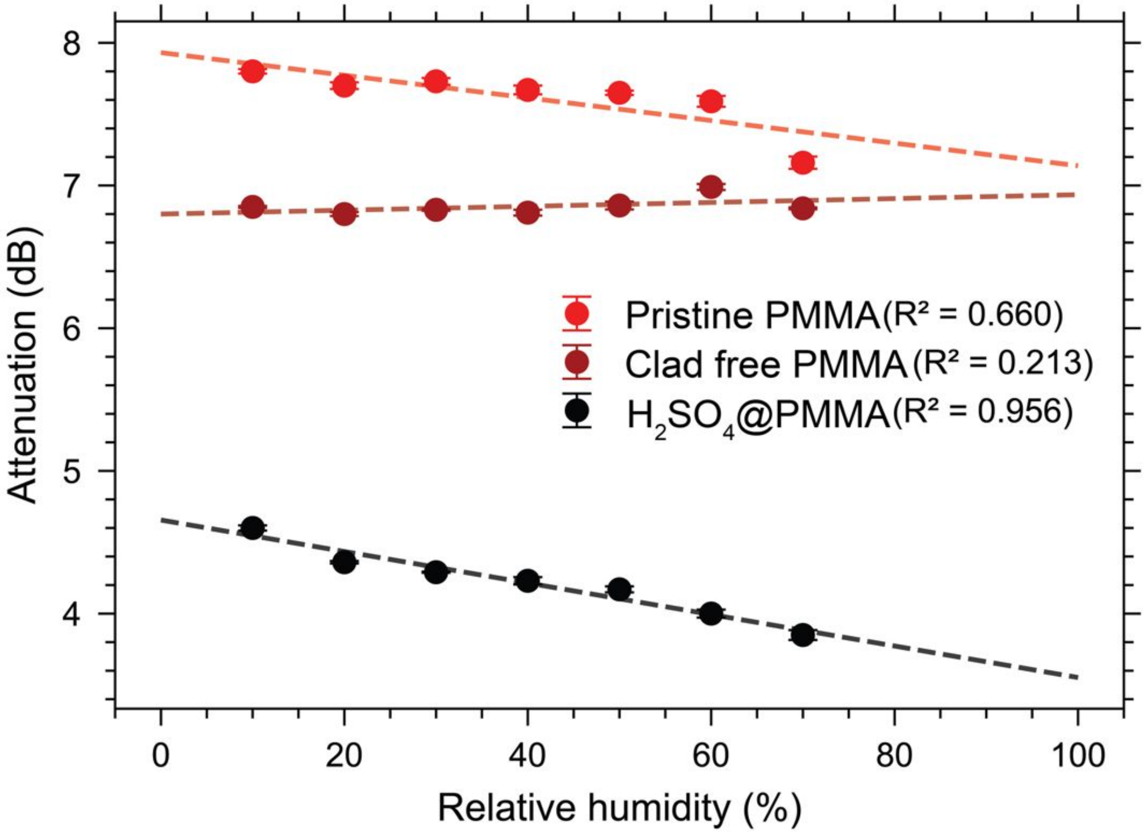

Figure S8. Attenuation as a function of %RH of PMMA optical fibers before and after chemical treatment at 520 nm.

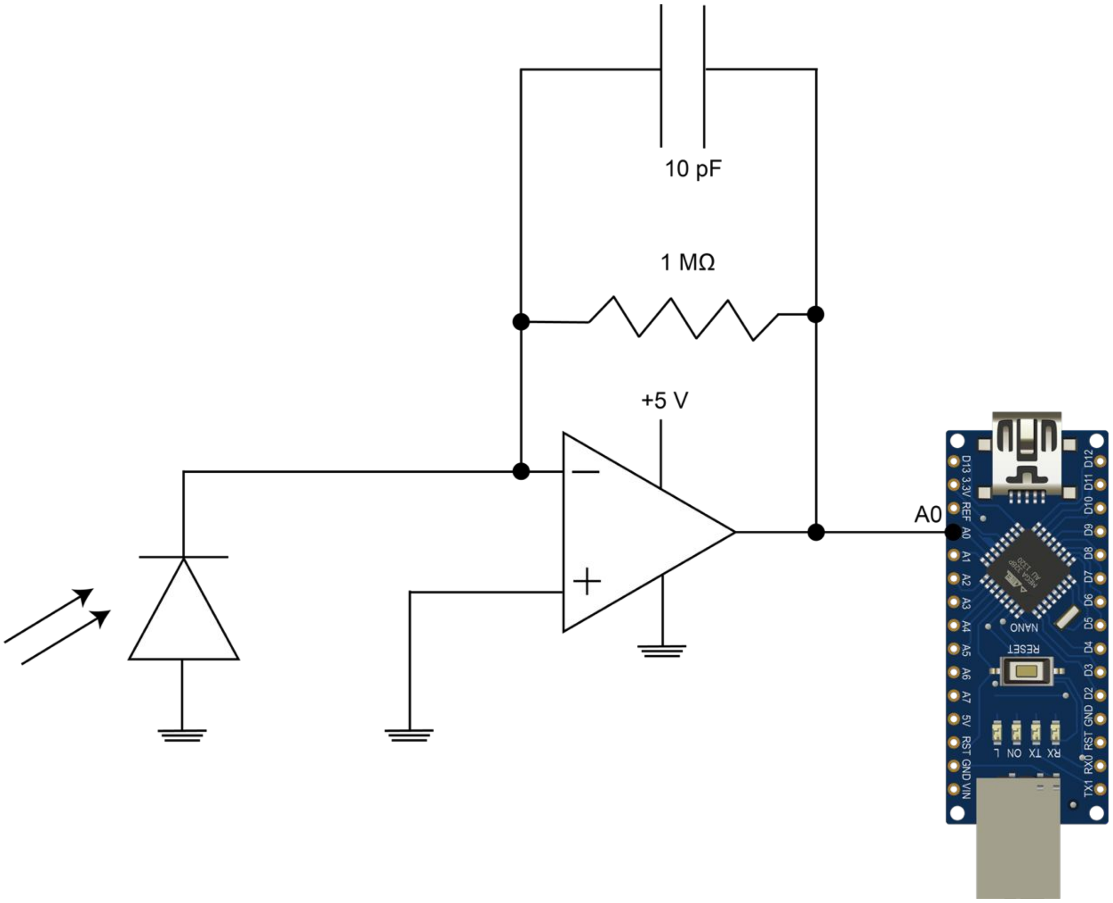

Figure S9. Schematic representation of the electronic circuit needed for making the sensor prototype.

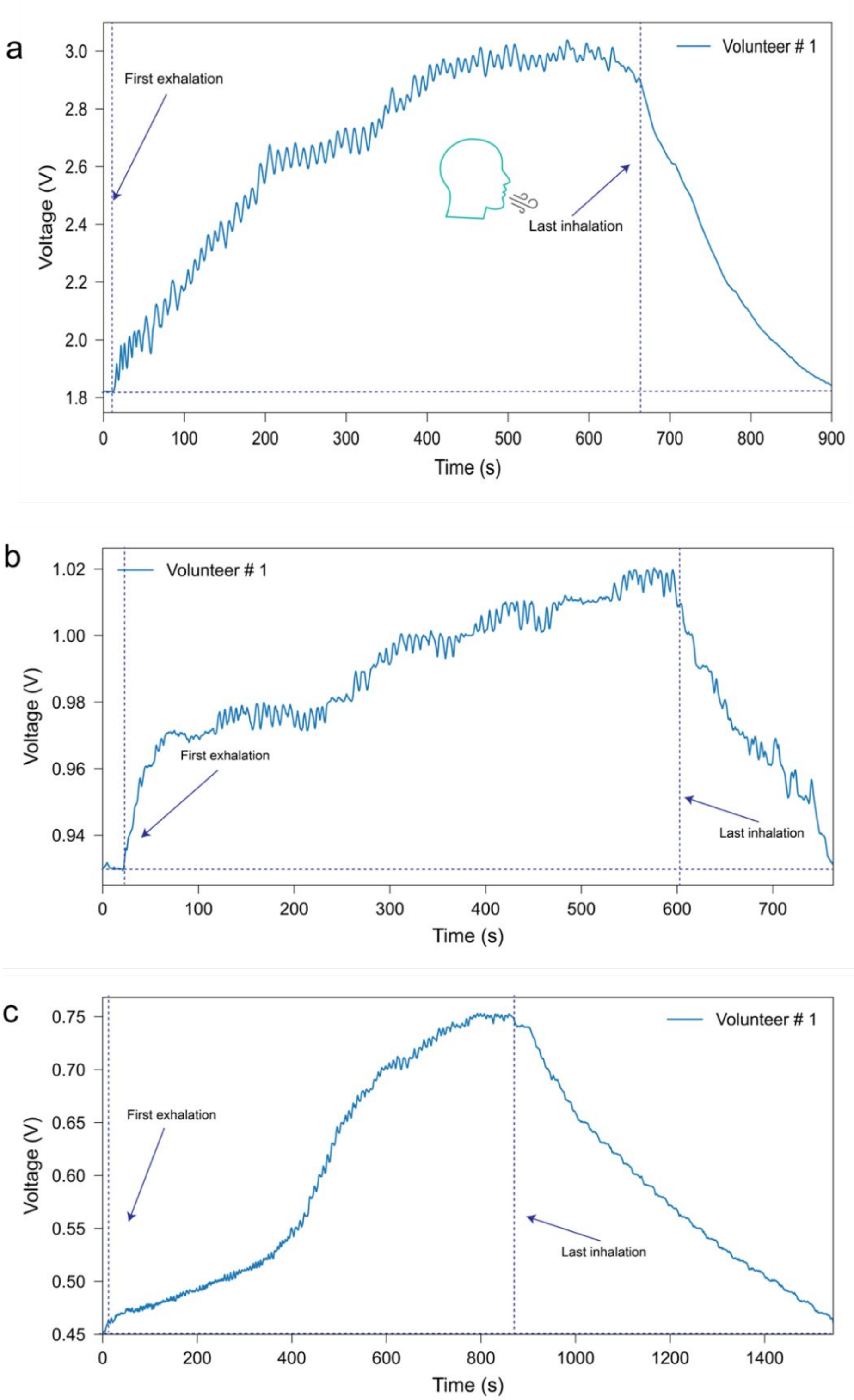

Figure S10. Choronovoltametry spectra were obtained through breathing on (a) Alg@PMMA-60, (b) Alg@PMMA-170, and (c) Alg@PMMA-370.

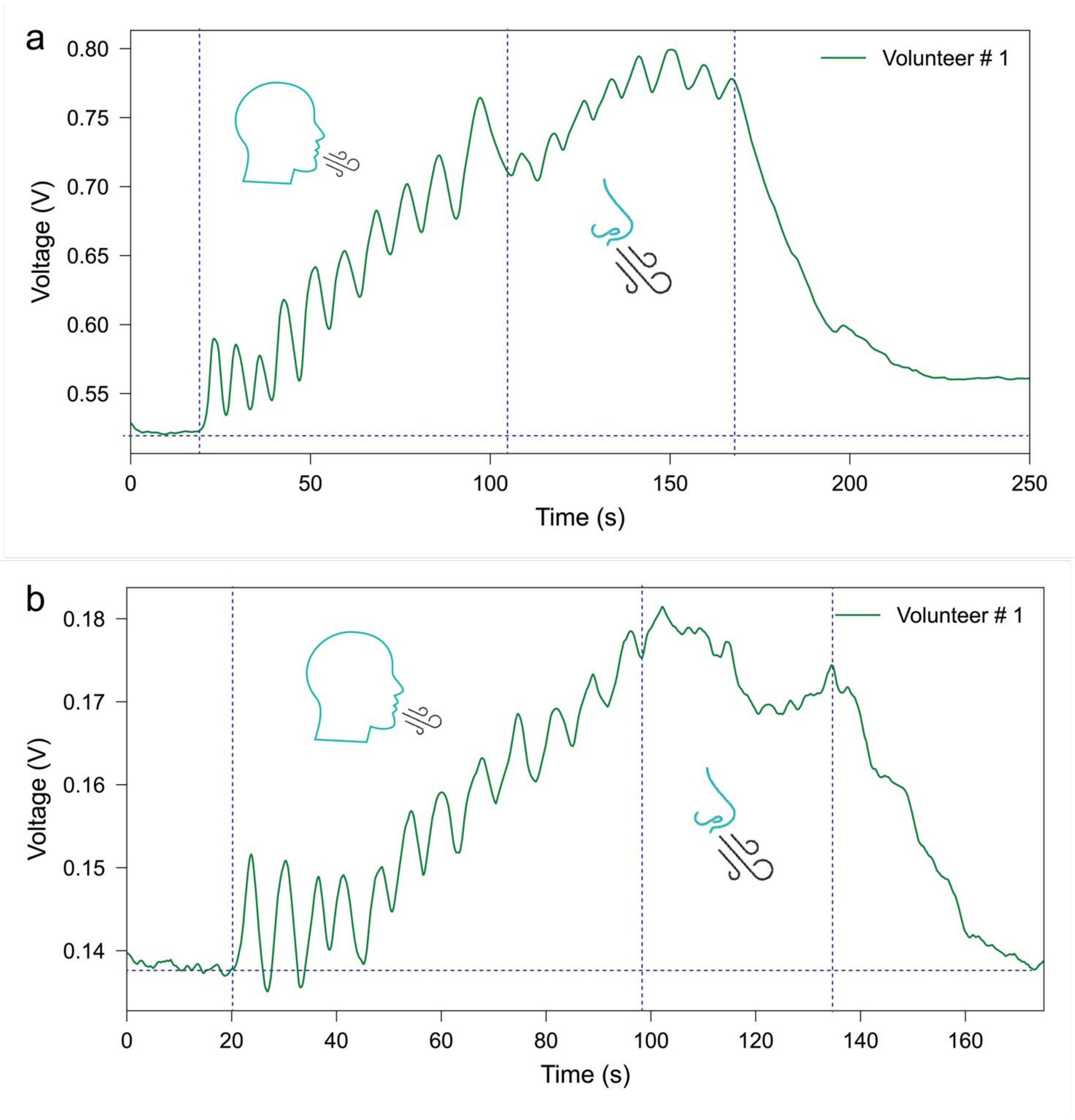

Figure S11. Chronovoltametry spectra of breathing response of Alg@PMMA-60 fiber using (a) red (640 nm) and (b) yellow LED (580 nm).

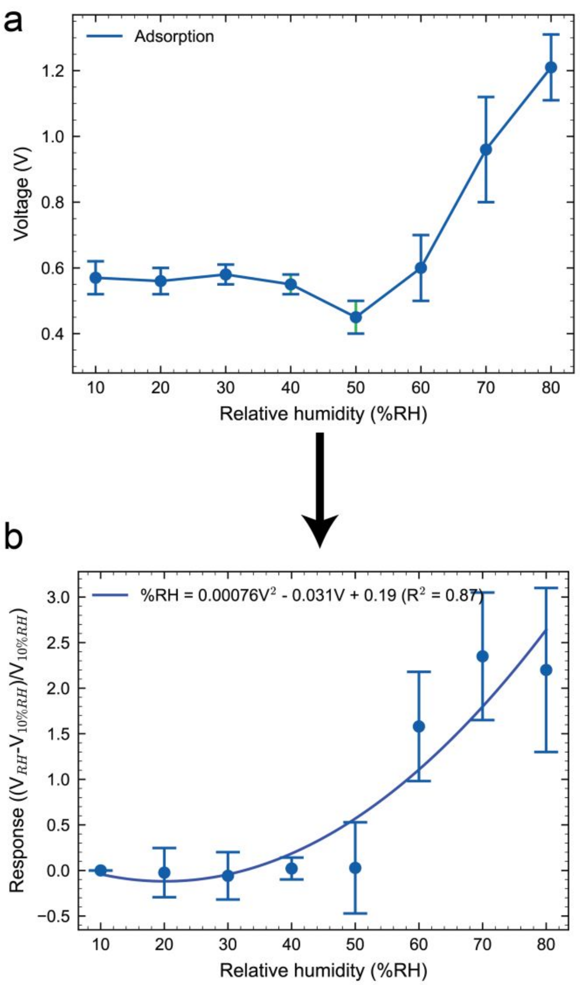

The output voltage across the alginate coated PMMA fiber in the presence of LED light source was measured an Arduino Nano at different relative humidity. A plot across relative humidity and output voltage is presented.

Response at specific output voltage was

$$\text{Response } ((V_{RH}-V_{10\%RH})/V_{10\%RH})$$

Where V_{RH} is the output voltage at specific relative humidity and $V_{10\%RH}$ is the output voltage at 10% RH. A polynomial fit was obtained to a correlation between voltage and relative humidity.

$$\%RH = 0.00076V^2 - 0.031V + 0.19$$

Figure S4. Humidity response and sensor calibration for Alg@PMMA-60 fiber integrated miniaturized sensor. (a) Measured voltage as a function of relative humidity (%RH) for absorption. (b) Sensor calibration curve at different humidity.

Table S1. Comparison of the properties of different fiber optic-based humidity sensors.

S.No	Material used	Humidity range (% RH)	Sensitivity	Response time (s)	Recovery time (s)	Reference
1.	Fluorinated cyclic transparent optical polymer (CYTOP)		-37 pm/%RH	N.A.	N.A.	1
2.	Gelatin coating optical fiber	70-90	-7.005 nm/%RH	N.A.	N.A.	2

3.	Polyvinyl alcohol coated tapered fiber	30-90	0.1194 nm/%RH			3
4.	Polyvinyl alcohol filled inside Fabry-Perot microcavity	46-75	248.9 pm/%RH	N.A.	N.A.	4
5	Chitosan filled inside microstructured optical fiber	30-95	68.5 pm/%RH	0.08	0.07	5
6	Gelatin coated microknot fiber	40-90	0.2 dB/%RH	0.084	0.029	6
7	Chitosan film as a Fabry-Perot sensing cavity	40-92	7.5 nm/%RH	0.978	0.686	7
8	Chitosan film and UVoptical adhesive composite coated on the tip of the fiber	45-90	1.45 nm/%RH	0.35	0.46	8
9	Agarose coated on POFs	20-80	0.41μW/%RH	1	4	9
10	Co-PANi coated PMMA optical fiber	20-100	3.4 mV/%RH	8	60	10
11	Polyimide film coated on the of a hollow-core fiber.	40-80	1.309 nm/%RH	4	4	11
12	Fabry-Perot optical fiber tip was coated with polyvinylidene fluoride	35-80	32.54 pm/%RH	-	-	12
13	Chitosan coated on the tip of Fabry-Perot optical fiber with custom microstructure	30-95	68.55 pm/%RH	0.08	0.07	13
14	Methylene-blue immobilized sol-gel coated fiber	1-70	0.087 dB/%RH	10	25	14

15	Alg@PMAA-60 integrated sensor	10-70	0.18 dB/%RH	1.3	1.3	This work

Table S2. Cost-estimate of components and materials.

S.No	Component name	Estimated price (USD)
1	Arduino Nano	10
2	TLC272BIP op-amp	0.5-1
3	1 MΩ resistor	0.1-0.25
4	10 pF capacitor	0.15-0.3
5	BPX 65 photodetector	2-5
6	Clear lens LED	0.25-0.5
7	Breadboard	2-5
8	9V battery	1-3
9	Jumper cable	2-5
10	PMMA optical fiber	1-5
11	Laser emitter module	24
	Total estimated cost	36-59

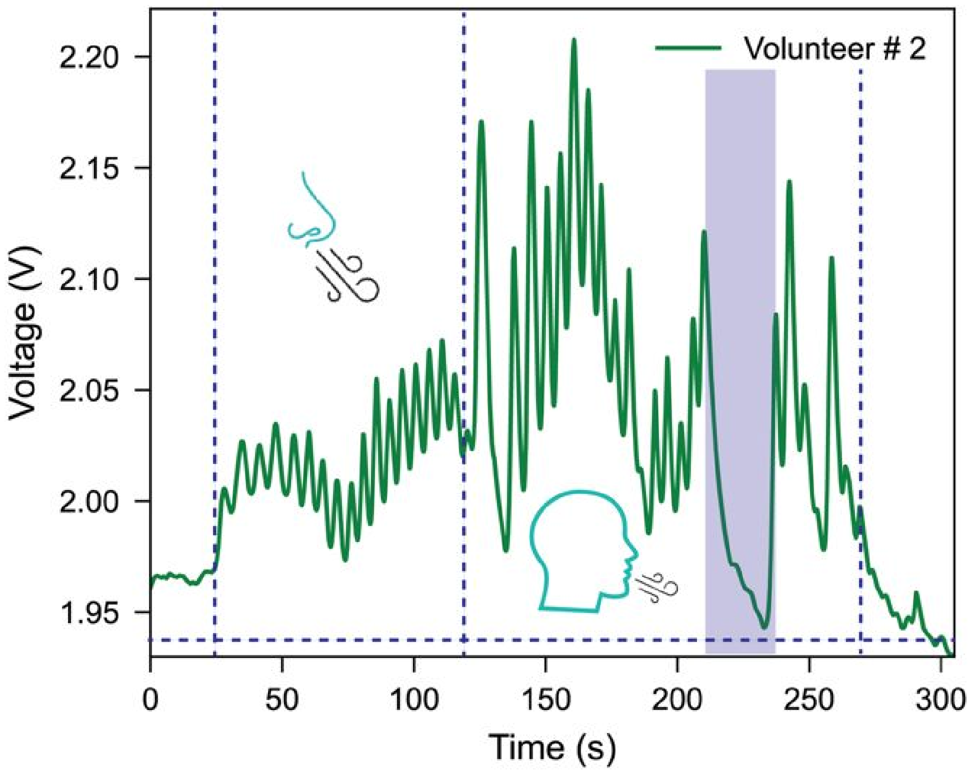

Figure S13. Chronovoltametric response of breathing under 930 nm LED.

References

(1) Theodosiou, A.; Kalli, K. Humidity Sensing Using Femtosecond Laser-Inscribed Fiber Bragg Gratings. *Opt. Fiber Technol.* **2020,** *54,* 102079.

(2) Yang, J.; Guan, C.; Yu, Z.; Yang, M.; Shi, J.; Wang, P.; Yang, J.; Yuan, L. A Flexible Humidity Sensor Based on Copper Nanosheets/Polyaniline Nanocomposites. *Sens. Actuators B Chem*. **2020**, *305*, 127555.

(3) Chen, N.; Zhou, X.; Li, X. High-Sensitivity Temperature Sensor Based on FBG and FPI Hybrid Structure. *IEEE Trans. Instrum. Meas.* **2021**, *70*, 1–8.

(4) Chen, M.; Zhao, Y.; Wei, H.; Zhu, C.; Krishnaswamy, S. Fast Response Optical Fiber Humidity Sensor with a Hollow Core Fiber Interferometer. *Sens. Actuators B Chem*. **2021**, *328*, 128981.

(5) Shrivastav, A. M.; Gunawardena, D. S.; Liu, Z.; Tam, H.-Y. Optical Fiber Sensor for Relative Humidity Measurement Using a Functionalized Coating. *Sci. Rep*. **2020**, *10*, 6002.

(6) Yi, Y.; Jiang, Y.; Zhao, H.; Brambilla, G.; Fan, Y.; Wang, P. Ultra-Sensitive Relative Humidity Sensor Based on Optical Microfiber. *ACS Sens*. **2020**, *5*, 3404–3410.

(7) Zhou, C.; Zhou, Q.; Wang, B.; Tian, J.; Yao, Y. Compact Fiber-Optic Humidity Sensor Using a Cascaded Fabry–Pérot Interferometer. *Opt. Express* **2021**, *29*, 11854–11868.

(8) Wang, Y.; Yan, Y.; Lian, Z.; Chen, D.; Lau, A. P. T.; Lu, C. All-Polymer Fabry–Pérot Interferometer for Ultrafast Humidity Sensing. *Opt. Express* **2022**, *30*, 39946–39960.

(9) Ghaffar, A.; Hussain, S.; Musavi, S. H. A.; Mehdi, M.; Zalkepali, N. U. H. H.; Lin, J.; Jianping, Y.; Chhattal, M.; Lan, K.; Cao, L. Flexible Humidity Sensor with Fast Response and Recovery Using Nanocomposite Film. *IEEE Sens. J.* **2024**, *24*, 7816–7823.

(10) Vijayan, A.; Fuke, M.; Hawaldar, R.; Kulkarni, M.; Amalnerkar, D.; Aiyer, R. C. PEDOT-PSS Based Humidity Sensor with Enhanced Sensitivity and Stability. *Sens. Actuators B Chem*. **2008**, *129*, 106–112.

(11) Bian, C.; Hu, M.; Wang, R.; Gang, T.; Tong, R.; Zhang, L.; Guo, T.; Liu, X.; Qiao, X. Optical Fiber Humidity Sensor with High Sensitivity Using Hydrophilic Coating. *Appl. Opt.* **2018**, *57*, 356–361.

(12) Vaz, A.; Barroca, N.; Ribeiro, M.; Pereira, A.; Frazão, O. Humidity Sensor Based on Hollow Core Fiber and Agarose Coating. *IEEE Photonics Technol. Lett*. **2019**, *31*, 549–552.

(13) Shrivastav, A. M.; Gunawardena, D. S.; Liu, Z.; Tam, H.-Y. Optical Fiber Sensor for Relative Humidity Measurement Using a Functionalized Coating. *Sci. Rep.* **2020**, *10*, 6002.

(14) Zhao, Z.; Duan, Y. A Wireless Passive Integrated Humidity Sensor Based on LTCC Technology and Its Application in Breath Monitoring. *Sens. Actuators B Chem*. **2011**, *160*, 1340–1345.
